# A dual-band four-port MIMO antenna with a partial ground plane for n257/n260/n261 band applications

**DOI:** 10.1038/s41598-026-43355-5

**Published:** 2026-03-11

**Authors:** Pankaj Kumar Gautam, Gunjan Srivastava, Dharmendra Kumar Jhariya, Akhilesh Mohan, Sachin Kumar, Sameena Pathan

**Affiliations:** 1 Department of Electronics and Communication Engineering, National Institute of Technology Delhi, Delhi, 110036 India; 2 Department of Electronics and Communication Engineering, Graphic Era (Deemed to be) University, Dehradun, 248002 India; 3 Department of Electronics and Communication Engineering, Indian Institute of Technology Roorkee, Roorkee, 247667 India; 4 Department of Electronics and Communication Engineering, Galgotias College of Engineering and Technology, Greater Noida, 201310 India; 5 Manipal Institute of Technology, Manipal Academy of Higher Education, Manipal, India

**Keywords:** Energy science and technology, Engineering

## Abstract

In this paper, a compact four-port dual-band multiple-input-multiple-output (MIMO) antenna is designed and developed for fifth-generation (5G) millimeter-wave applications. The proposed antenna operates in the n257 band at 28 GHz and the n260/n261 bands at 38 GHz within the 5G new radio (NR) frequency range-2 (FR2) spectrum, thereby supporting enhanced data rates, high capacity, and low-latency applications. The dual-band unit element of the proposed 4-port MIMO antenna consists of a modified rectangular patch antenna with a partial ground plane. The modified patch structure is obtained by loading a microstrip-fed rectangular patch with H-shaped and inverted T-shaped slots. The unit elements are placed orthogonally to each other, and they are interconnected at the center via extended stubs. The designed dual-band MIMO antenna has an overall volume of 28 mm × 28 mm × 0.254 mm, which is approximately $0.161{\boldsymbol{\lambda}}_{0}^{3}$, where ${\boldsymbol{\lambda}}_{0}$ corresponds to the operating wavelength at 28 GHz. The designed MIMO antenna operates at 27.26–29.90 GHz and 37.57–40.29 GHz, which fall within the n257/n261 and n260 bands of the 5G NR FR2 spectrum, with inter-port isolation better than 20 dB across both bands. The proposed antenna system demonstrates excellent MIMO performance, validating its ability to support reliable, high-capacity, and low-latency communication, making it a strong candidate for next-generation 5G millimeter-wave applications .

## Introduction

The fifth generation (5G) communication technology is being popularly used nowadays due to its numerous advantages, such as larger bandwidth, low latency, and high data rates, which make it suitable for augmented reality, robotics, and internet of things (IoT) applications^1–3^. For the millimeter-wave spectrum, 28 GHz, 38 GHz, 60 GHz, and 73 GHz frequency bands are allocated for wireless networks, and their radio propagation models are presented^4^. The lower millimeter-wave frequencies, 28 GHz and 38 GHz, are preferred over higher ones (60 GHz, 73 GHz) due to their low atmospheric absorption, which leads to lower path losses^5,6^. Several 5G communication systems operating either in 28 GHz or in 38 GHz are reported in the literature^7–11^. In^7^, a compact Y-shaped patch antenna operating in 27.5–28.25 GHz frequency band is presented. A multilayer high-gain (~ 13.9 dBi) 5G antenna with near zero refractive index metamaterial for 27–29 GHz frequency spectrum is presented^8^. In^9^, an elliptical slot excited elliptical patch antenna for 28 GHz with 10 dBi gain is reported. A patch antenna with defected ground structure (DGS) for 26.5–32.9 GHz frequency spectrum is designed^10^. In the design, the ground plane defects are used to achieve wideband operation. In^11^, an ultra-compact arc-shaped elliptical slot patch antenna for 25.83–30.24 GHz frequency band is reported.

Multifunctional communication systems are widely used, requiring dual-band antennas because modern wireless devices must support multiple standards and services that operate at different frequency bands. Using a single dual-band antenna reduces size, cost, and hardware complexity while ensuring compatibility, spectrum efficiency, and seamless connectivity across various communication networks. Several methodologies have been reported in the literature for designing and developing dual-band antenna systems for the lower millimeter-wave frequency spectrum^12–15^.

In recent years, multiple-input-multiple-output (MIMO) antenna systems, which deploy multiple antennas at both the transmitter and receiver and operate in the millimeter-wave spectrum, have gained popularity due to their ability to deliver higher data rates, enhanced channel capacity, improved spectral efficiency, and reduced multipath fading. Numerous studies on 2-element and 4-element dual-band MIMO antennas at millimeter-wave (28/38 GHz) for 2 × 2 and 4 × 4 MIMO systems have been conducted in the open literature^16–22^. In^16^, a two-port dual-band composite patch MIMO antenna with parasitic patches for the lower millimeter-wave frequency spectrum (28/38 GHz) is reported, which have the impedance bandwidths of 1.23 GHz and 1.06 GHz, respectively. In^17^, a high-profile dual-band two-port MIMO antenna operating at 26/38 GHz is presented, incorporating a 3D-printed dielectric lens to achieve enhanced gain for 5G IoT applications. In^18^, a dual-band composite patch of circular and elliptical structures four-element MIMO antenna is presented. The designed MIMO antenna operated in 27.76–28.48 GHz (BW ≈ 0.72 GHz) and 37.69–38.19 GHz (BW ≈ 0.50 GHz) frequency bands with the inter-port isolations better than 18 dB and 22 dB, respectively. In^19^, dual-band four-element MIMO antennas consisting of two interconnected patches for 28 GHz (BW ≈ 0.60 GHz) and 38 GHz (BW ≈ 0.60 GHz) frequencies are designed with inter-ports isolations better than 30 dB across both the operating bands. Another two interconnected patch four-port MIMO antenna at 28 GHz (BW ≈ 2.7 GHz) and 38 GHz (BW ≈ 0.75 GHz) is reported^20^. In these designs, the microstrip-fed patch acts as the primary radiator, while another parasitic patch acts as the secondary radiator. Here, the decoupling network consisting of a circular ring and four copper strips is also deployed along with a modified ground plane to achieve inter-port isolations better than 30 dB. In^21^, a four-port dual-band 28 GHz/38 GHz MIMO antenna with impedance bandwidths of 0.718 GHz and 0.56 GHz is reported. It incorporates four orthogonally arranged modified planar patches. The four modified patches are placed half-wavelength apart, and an additional electromagnetic bandgap (EBG) structure is placed among them to reduce the mutual couplings. In^22^, a 28/38 GHz four-port cross-shaped MIMO antenna operating at 27.5–29.3 GHz (BW ≈ 1.80 GHz) and 37.5–38.2 GHz (BW ≈ 0.70 GHz) frequency bands is presented for 5G applications. The designed antenna incorporates three triangular slots in a rectangular patch to enhance impedance matching. An inter-port isolation better than 20 dB is obtained by placing a thin parasitic strip is between the two elements.

From the review of the aforementioned literature, several notable limitations are observed: (i) the designs presented in^16–22^, have lesser operating bandwidths in both the operating bands. The larger bandwidths are essential in multiband MIMO antennas to support high data rates and accommodate wide communication channels. (ii) The designs presented in^16–22^, have lower inter-port isolations across both operating bands. High inter isolations ensure independent signal transmission and reception at each port, leading to improved diversity gain, higher channel capacity, lower envelope correlation coefficient (ECC), and more reliable overall MIMO performance across all operating frequency bands. (iii) The designs reported in^17,18^ have complex designs. Simpler designs are preferred as they reduce fabrication complexity, cost, and potential losses, while improving reliability and ease of integration. The main challenges in previously reported dual-band millimeter-wave MIMO antenna systems include achieving a simple structure, low-profile configuration, wide bandwidth, and high inter-port isolation simultaneously. Integrating all these features within a single dual-band MIMO antenna design remains a significant challenge for antenna designers.

To overcome these challenges, a four-element dual-band millimeter-wave MIMO antenna with a partial ground plane is designed and developed in this paper. The unit element is designed using a modified rectangular patch antenna, enabling operation in the 28 GHz and 38 GHz millimeter-wave bands. The modified patch structure is obtained by loading a microstrip-fed rectangular patch with an H-shaped and inverted T-shaped slot. A partial ground plane creates an alternative current pathway distinct from that of the radiating element. While the patch predominates at higher frequencies, currents may flow mostly on the modified ground-plane structure at lower frequencies. The unit elements are placed orthogonally to each other, and they are interconnected at the center via extended stubs. The designed MIMO antenna operates in the 27.26–29.90 GHz and 37.57–40.29 GHz bands, which fall within the n257/n261 and n260 bands of the 5G new radio (NR) frequency range-2 (FR2) spectrum, with inter-port isolation better than 20 dB across both operating bands. The designed MIMO antenna also exhibits peak realized gains of 6.41 dBi and 8.50 dBi in the lower and higher operating bands, respectively.

**Fig. 1:**
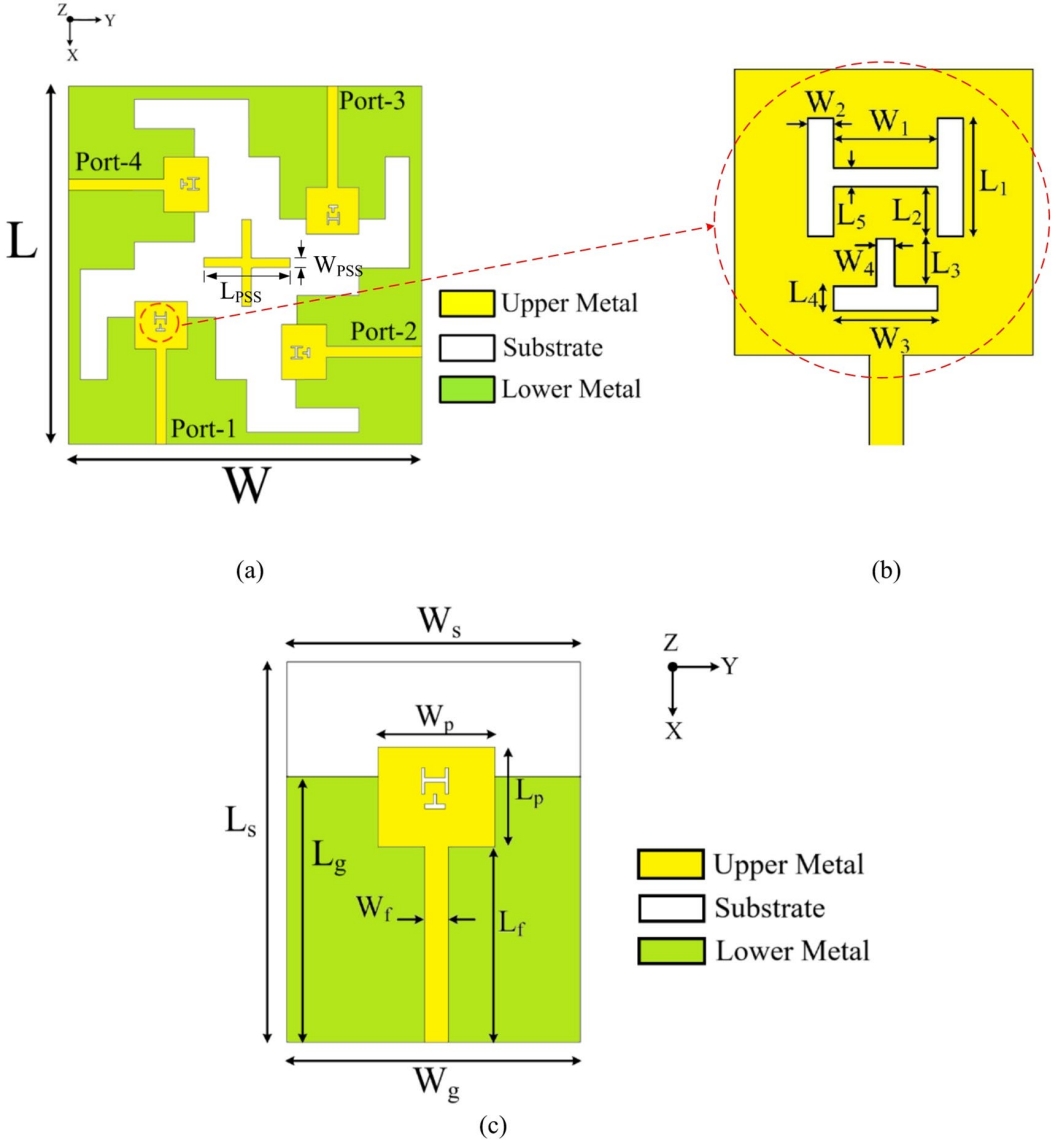
Geometric layout: (**a**) Proposed MIMO antenna, (**b**) Magnified view of the modified patch, (**c**) Unit antenna element [Dimensions (in mm): *W* = 28, *L* = 28, *W*_*s*_ = 10, *L*_*s*_ = 15.33, *W*_*p*_ = 5, *L*_*p*_ = 4.6, *W*_*f*_ = 0.78, *L*_*f*_ = 7.83, *W*_1_ = 1.4, *W*_2_ = 0.2, *W*_3_ = 1.4, *W*_4_ = 0.15, *L*_1_ = 1.4, *L*_2_ = 0.7, *L*_3_ = 0.8, *L*_4_ = 0.2, *L*_5_ = 0.15, *L*_g_ = 10.83, *W*_g_ = 10, *L*_*PSS*_ = 6.3, *W*_*PSS*_ = 0.65].

The key contributions of the proposed work are as follows:

(i) It is the first instance when the partial ground plane is utilized for the design of dual band millimeter-wave MIMO antenna.
(ii) The designed MIMO antenna possesses dual-band functionality at 28 GHz (n257/n261) and 38 GHz (n260) for 5G NR FR2 applications.
(iii) The designed MIMO antenna possess wide impedance bandwidth in both 27.26–29.90 GHz (BW ≈ 2.64 GHz) and 37.57–40.29 GHz (BW ≈ 2.72 GHz) frequency bands.
(iv) It possesses the volume of 2.61*λ*_0_ × 2.61*λ*_0_ × 0.0237*λ*_0_ at 28 GHz (28 mm × 28 mm × 0.254 mm) and inter-port isolation greater than 24 dB across both bands, minimizing mutual coupling.

### Antenna configuration

Figure 1 illustrates the geometric layout of the proposed dual-band four-port MIMO antenna along with its dimensions. It is designed on a low-loss, high-frequency Rogers RT/Duroid 5880 laminate with a relative permittivity of 2.2 and a loss tangent of 0.0008. The proposed dual-band MIMO antenna features a compact footprint of *L* × *W* and a substrate thickness of 0.254 mm, enabling efficient integration into space-constrained high-frequency wireless systems. The proposed MIMO antenna consists of four dual-band modified rectangular patch elements [Fig. 1(a)], each excited by a microstrip feed and arranged orthogonally. The unit element achieves dual-band operation by incorporating an H-shaped slot and an inverted-T-shaped slot [Fig. 1(b)] into the rectangular patch. The partial ground planes of the individual antenna elements are extended toward the center [Fig. 1(c)], forming a plus shaped structure (PSS) that enhances the isolation of the proposed MIMO antenna. The interconnected ground plane not only reinforces the antenna’s structural integrity but also ensures a uniform ground reference across all ports, thereby enhancing signal stability and port-to-port isolation. In this work, electromagnetic simulations are performed using the ANSYS HFSS v21 3D EM solver.

## Design methodology

This section thoroughly examines the unit element design and the operation of the proposed MIMO system.

### Unit element

**Fig. 2:**
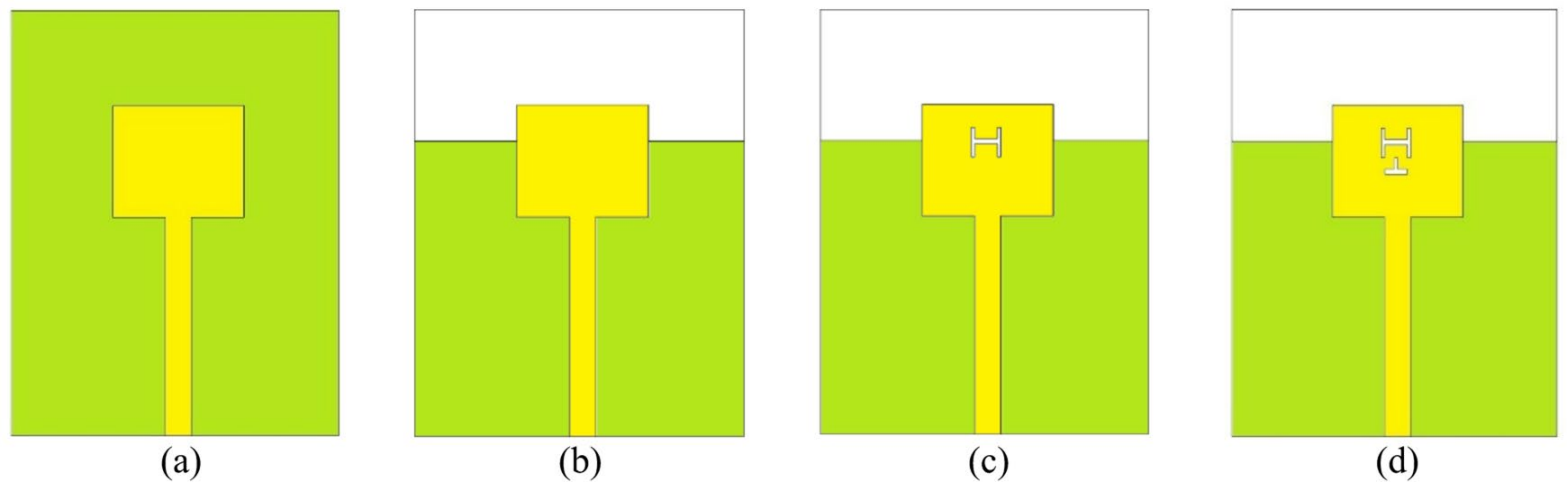
Design stages of the proposed antenna for dual-band (28/38 GHz) operation: (**a**) Stage-I, (**b**) Stage-II, (**c**) Stage-III, (**d**) Stage-IV.

**Fig. 3:**
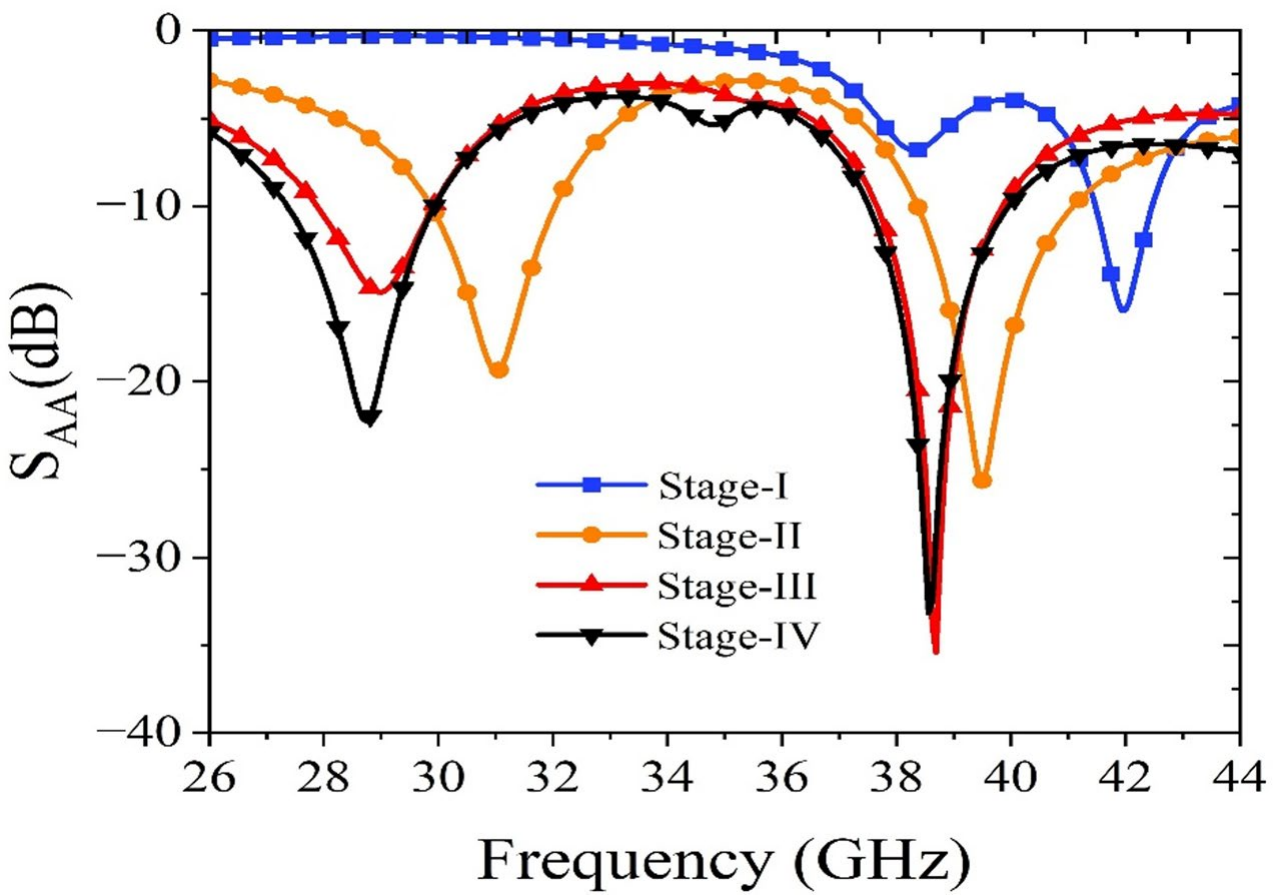
*S*_*AA*_ plot of the stage-wise unit element antenna.

**Fig. 4:**
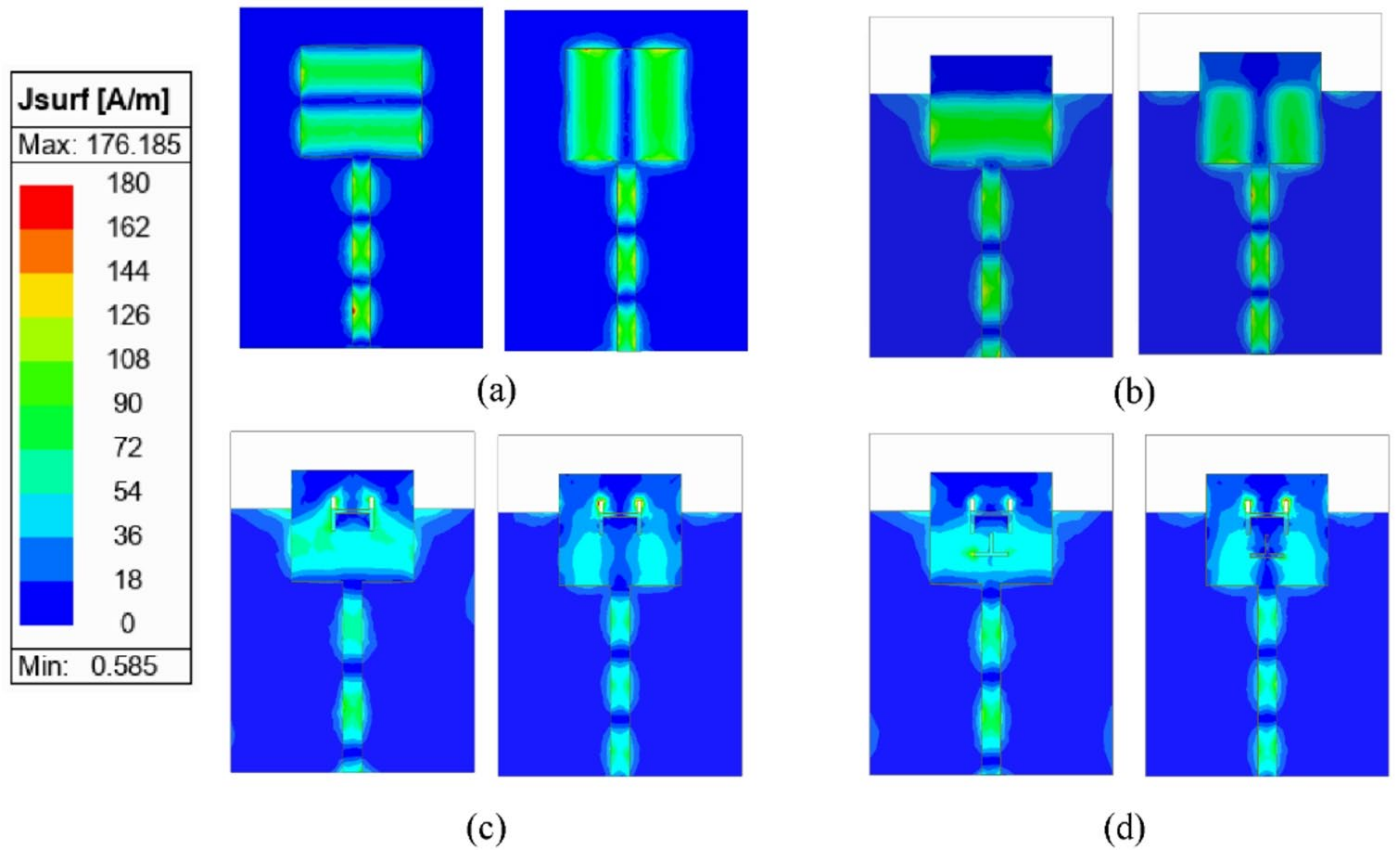
Simulated surface current distributions of different design stages of the proposed unit element antenna at 28/38 GHz: (a) Stage-I, (b) Stage-II, (c) Stage-III, (d) Stage-IV.

The step-by-step evolution of the unit element used in the proposed MIMO antenna is illustrated in Fig. 2, and the corresponding *S*_*AA*_-parameters are plotted in Fig. 3. Initially, in Stage-I [Fig. 2a], the antenna design consists of a simple rectangular patch of dimensions *L*_*p*_ × *W*_*p*_ backed by a full ground plane of dimensions *L*_*g*_ × *W*_*g*_. This configuration resonates at 38 GHz and 42 GHz, as shown in Fig. 3. The surface current distributions at 38 GHz and 42 GHz are represented in Fig. 4a. It can be observed from the surface current distributions that the unit element in Stage-I utilizes TM_02_ and TM_20_ modes at 38 GHz and 42 GHz, respectively.

To shift the resonant frequencies of the Stage-I unit element, the ground plane is truncated to form a partial ground configuration. Compared to a conventional full ground plane, this modification significantly alters the electromagnetic behavior of the antenna. The truncation increases fringing fields at the ground edge, which effectively increases the electrical length of the structure and modifies the effective dielectric loading. Consequently, the resonant frequencies shift, typically toward lower values. In addition to frequency tuning, the partial ground plane perturbs the surface current distribution and introduces additional current paths as shown in Fig. 4b. In Stage-II [Fig. 2b], this partial ground configuration enables dual-band operation at 31 GHz and 40 GHz, as illustrated in Fig. 3.

In Stage III [Fig. 2c], an H-shaped slot is introduced into the rectangular patch to achieve dual-band operation at 29 GHz and 38 GHz. The etched slot perturbs the current distribution, shifting the resonant frequencies. Furthermore, to enhance impedance matching at 28 GHz, an inverted T-shaped slot is incorporated into the rectangular patch, which introduces additional capacitance, causing a slight shift in the resonance without changing the physical dimensions of the rectangular patch. The antenna element developed in Stage IV [Fig. 2d] serves as the unit element for the proposed MIMO antenna system. This antenna element exhibits dual-band radiation characteristics, operating efficiently at 28 GHz and 38 GHz [Fig. 3]. The surface current distributions at Stage III and Stage IV are represented in Fig. 4c and Fig. 4d, respectively.

### Proposed dual-band MIMO antenna

**Fig. 5:**
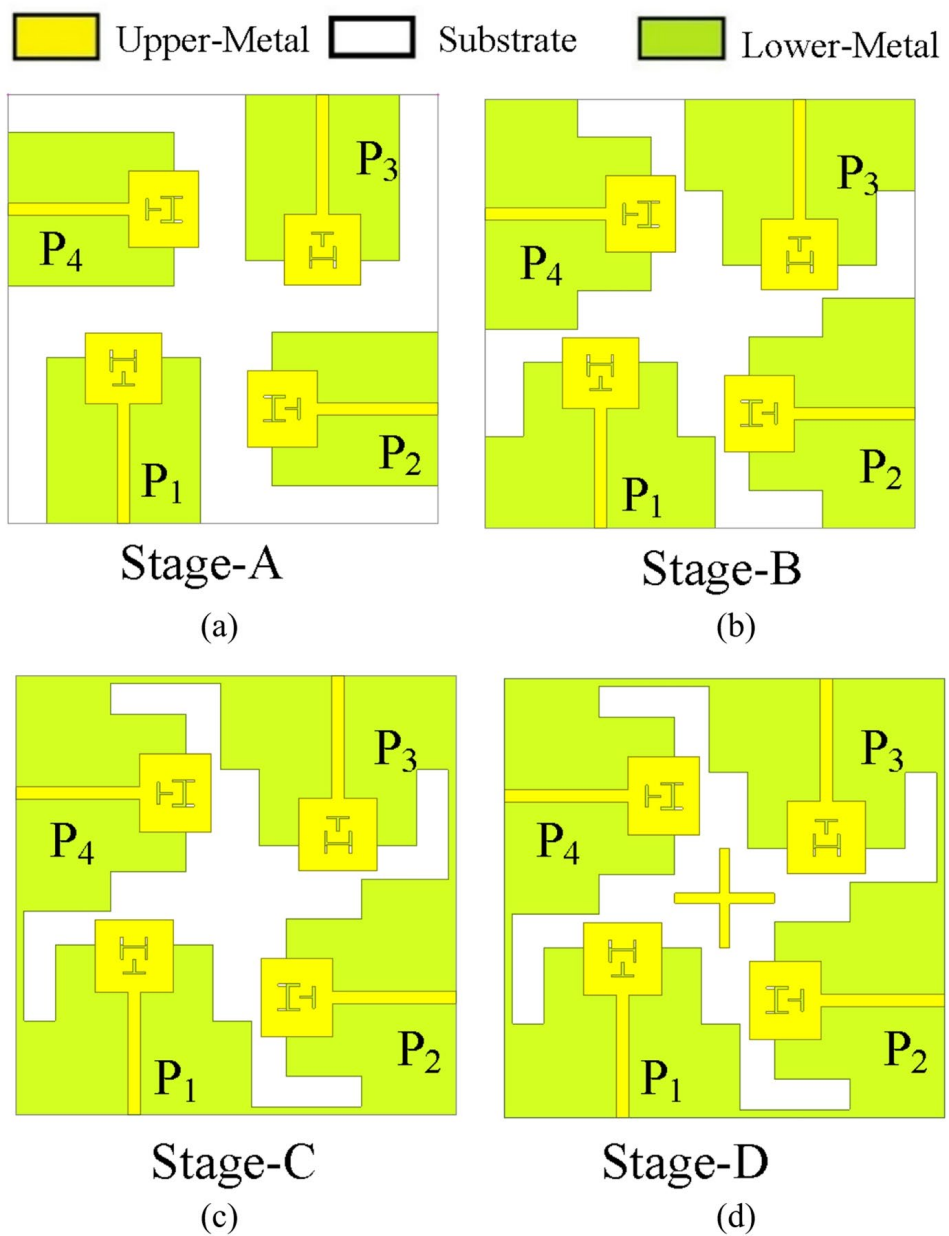
Evolution of the proposed four-port dual-band MIMO antenna: (**a**) Stage-A, (**b**) Stage-B, (**c**) Stage-C, (**d**) Stage-D.

**Fig. 6:**
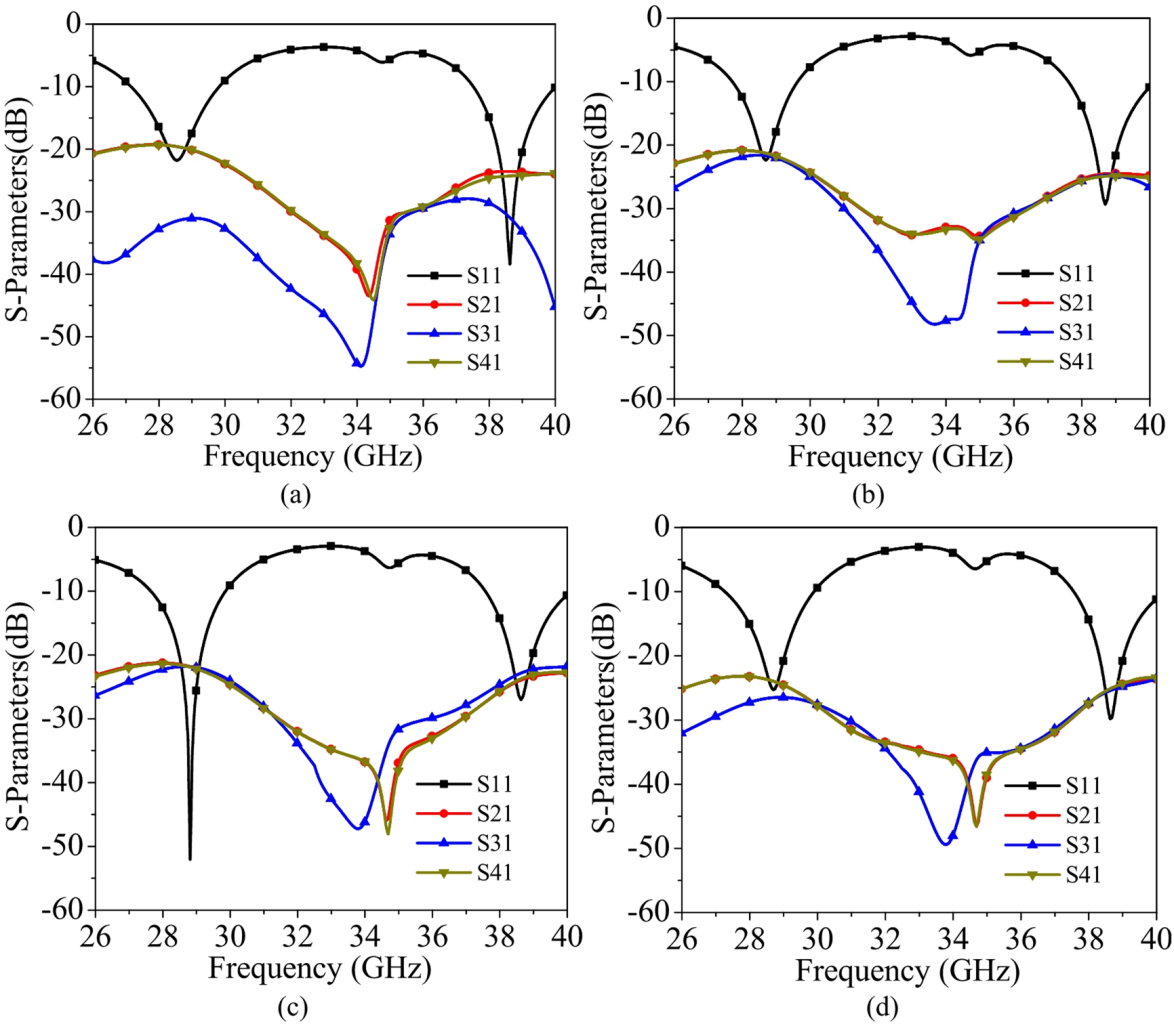
Simulated S-parameters of different evolution stages: (**a**) Stage-A, (**b**) Stage-B, (**c**) Stage-C, (**d**) Stage-D.

To realize a four-port dual-band MIMO antenna operating at 28 GHz and 38 GHz, the unit elements developed in the previous section are arranged orthogonally to each other, as depicted in Fig. 5(a) [Stage-A]. Orthogonal placement not only allows for the compact integration of multiple antenna elements within a small area, but it also helps to minimize mutual coupling between adjacent elements. Such an arrangement is crucial for achieving good isolation and reliable performance in MIMO systems, especially in high-frequency applications where spatial constraints and interference mitigation are significant design considerations. The MIMO antenna in Stage-A radiates at 28 GHz and at 38 GHz with inter-port isolations better than 18 dB across both the bands [Fig. 6(a)]. Since the width of the ground plane for each individual unit element was initially 10 mm, it was insufficient to properly accommodate the Rosenberger 02K243-40ME3 end-launch connectors, leading to potential mechanical instability and inadequate RF grounding at the feed interface. To ensure reliable connector attachment, the ground plane has been extended by 3 mm on both sides in Stage-B [Fig. 5(b)]. The MIMO antenna in Stage-B radiates at 28 GHz and at 38 GHz with inter-port isolations better than 20 dB across both the bands.

Further, the ground planes of each orthogonally placed antenna unit elements are interconnected by 0.5 mm wide metallic strips along their edges, forming a continuous, unified ground structure, as illustrated in Stage-C [Fig. 5c]. The connected geometry ensures structural integrity and contributes to consistent ground reference across all ports. It radiates at 28 GHz within the frequency range 27.65–29.89 GHz, providing an impedance bandwidth of 1.24 GHz, and at 38 GHz within the frequency range 37.57–40.14 GHz, offering an impedance bandwidth of 2.57 GHz. It also achieves inter-port isolation levels of better than 20 dB in both the lower and upper bands, ensuring minimal mutual coupling between the antenna elements. To ensure robust MIMO performance, inter-port isolation levels must be maintained above 20 dB across the entire operating bandwidth, thereby minimizing mutual coupling and enhancing overall system efficiency. Furthermore, to enhance inter-port isolation between the antenna elements and improve impedance bandwidth at both resonant frequencies, two rectangular strips of length *L*_*PSS*_ are placed to form a plus-shaped structure (PSS) at the center of the MIMO design, as illustrated in Stage-D [Fig. 5d]. The Stage-D is the proposed four-port MIMO antenna, with a plus-shaped structure that serves as a neutralization and current redistribution element.

When one port is excited, strong surface currents are induced on the common ground plane and tend to couple to adjacent elements through near-field interaction. The centrally placed orthogonal strips provide an additional coupling path with opposite-phase induced currents, thereby cancelling part of the mutual coupling between the ports. Consequently, Stage-D (the proposed four-port MIMO antenna) achieves better isolation and wider impedance bandwidth compared to the previous design stages. Thus, the proposed MIMO antenna operates within two frequency bands: 27.26–29.90 GHz, offering an impedance bandwidth of 2.64 GHz around 28 GHz, and 37.57–40.29 GHz, providing an impedance bandwidth of 2.72 GHz near 38 GHz [Fig. 6(d)]. The incorporation of the plus-shaped structure at the center of the MIMO design effectively improves inter-port isolation by approximately 20%, resulting in isolation levels exceeding 24 dB across the operating frequency bands.

### Role of plus shaped structure (PSS) in isolation mechanism

**Fig. 7:**
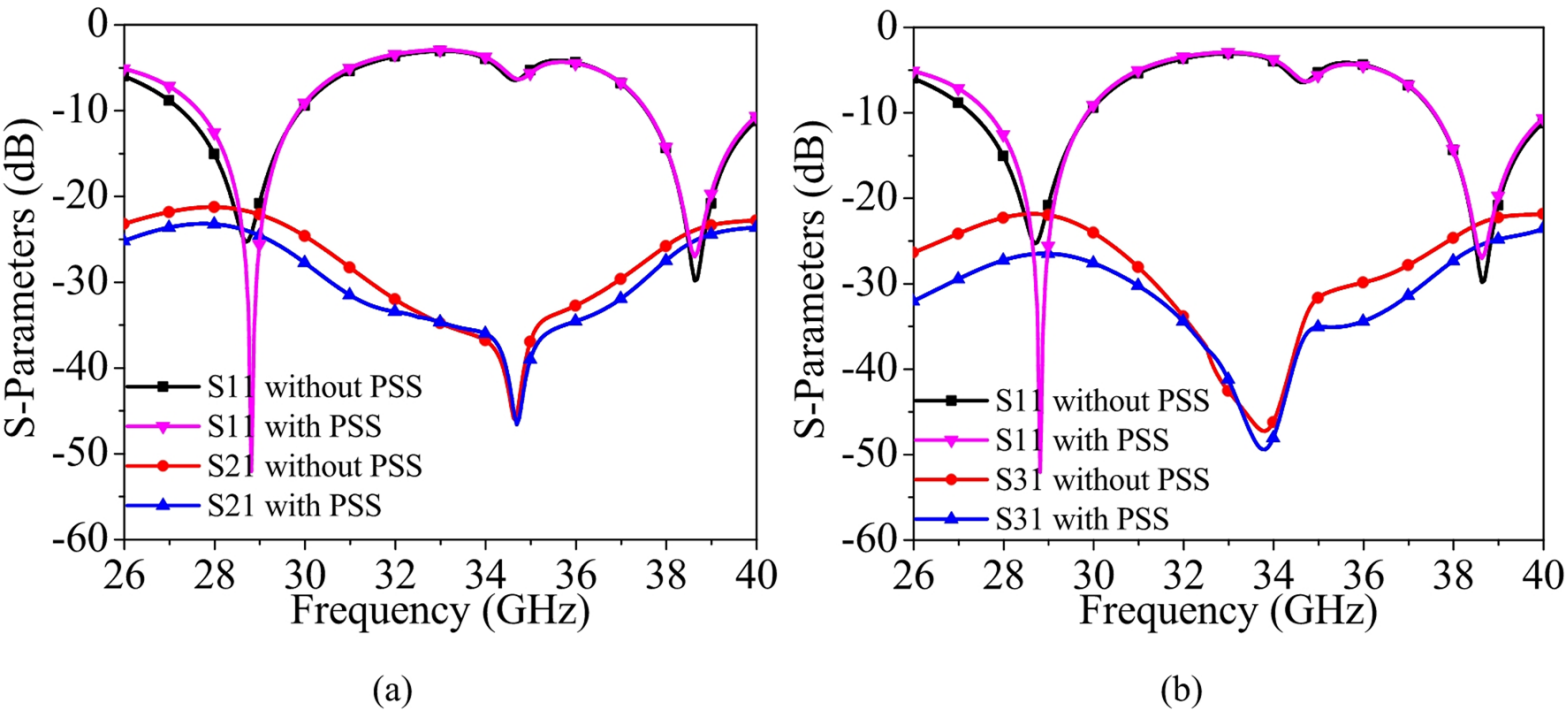
Simulated S-parameters without and with PSS: (**a**) S_21_, (**b**) S_31_.

A PSS placed between antenna elements in a MIMO configuration reduces mutual coupling primarily by disturbing and redistributing the surface currents flowing on the ground plane. When one antenna element is excited, strong surface currents are induced on the ground, and these currents can easily propagate toward adjacent elements, causing unwanted electromagnetic coupling. The centrally positioned PSS intercepts these currents and modifies their path, effectively acting as a perturbation element. This interruption reduces the magnitude of induced currents on neighboring elements, thereby lowering the mutual coupling and improving the inter-port isolation. Figure 7 shows the simulated S-parameters of the proposed MIMO antenna without and with the PSS. It is also evident from the figure that the introduction of the PSS structure enhances the impedance bandwidth by nearly 500 MHz (~1.8%) at the lower operating frequency of 28 GHz, while the impedance bandwidth at the higher band of 38 GHz remains almost unchanged. The inter-port isolation between Port 1 and Port 2 improves by 4 dB (~20%) and 3 dB (~15%) in the lower and upper operating bands, respectively. Similarly, the isolation between Port 1 and Port 3 increases by 8 dB (~40%) at the lower band and 5 dB (~20%) at the upper band.

**Fig. 8:**
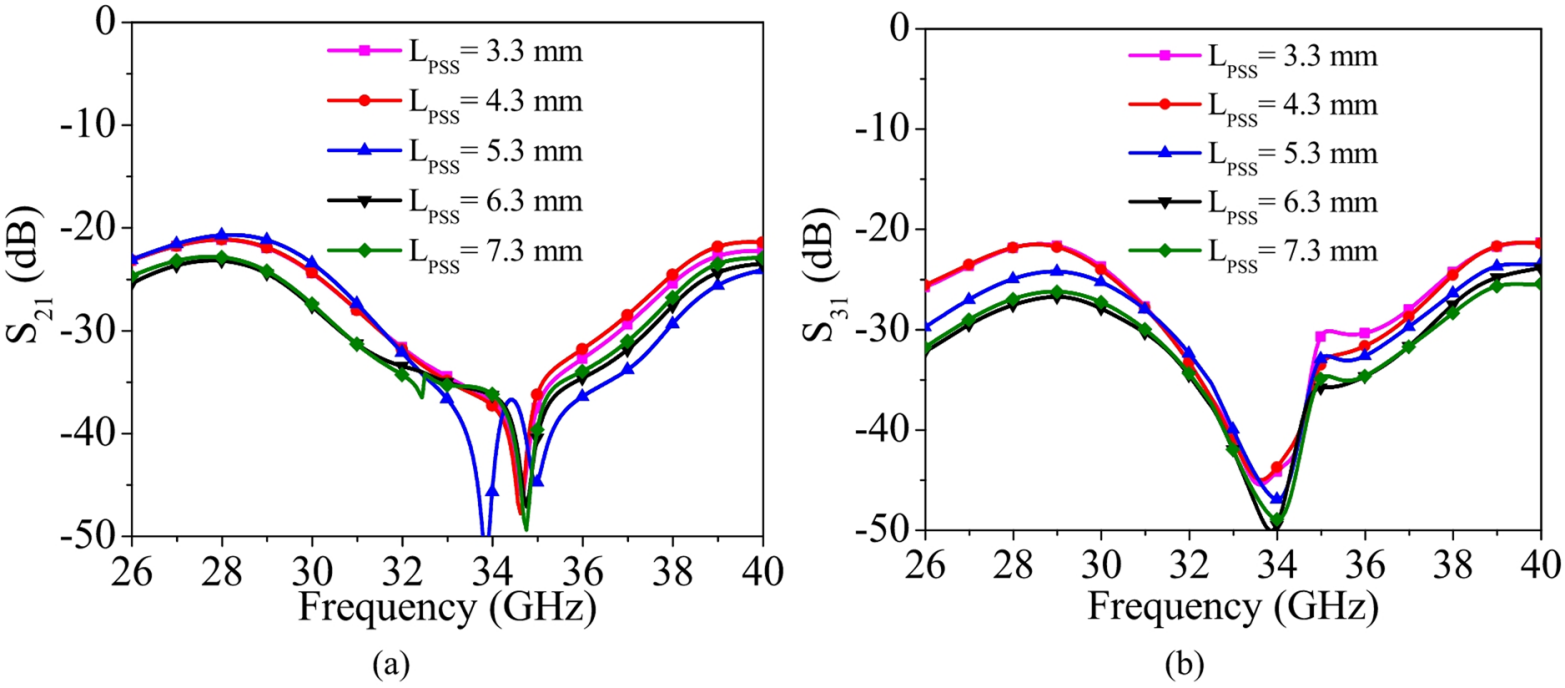
Simulated S-parameters for different values of *L*_*PSS*_: (a) S_21_, (b) S_31_.

To gain a clearer understanding of the isolation enhancement mechanism, a parametric analysis was carried out for the design parameter *L*_*PSS*_​ of the PSS. Figure 8 illustrates the simulated inter-port isolations, S_21_​ and S_31_​, for different values of *L*_*PSS*_​. The parameter *L*_*PSS*_ is varied from 3.3 mm to 7.3 mm in steps of 1 mm to investigate its influence on coupling behaviour. It is observed that as *L*_*PSS*_ ​ increases from 3.3 mm to 6.3 mm, both S_21_​ and S_31_​ progressively improve, indicating enhanced suppression of mutual coupling. This improvement can be attributed to the increased effective electrical length of the PSS, which strengthens its current-blocking and field-cancellation effects. However, when *L*_*PSS*_ is further increased to 7.3 mm, the inter-port isolation deteriorates. This degradation occurs because excessive length alters the resonance condition of the PSS, leading to impedance mismatch or reduced decoupling efficiency. Therefore, *L*_*PSS*_ = 6.3 mm is selected as the optimal design parameter, providing the best trade-off between coupling suppression and impedance performance.

**Fig. 9:**
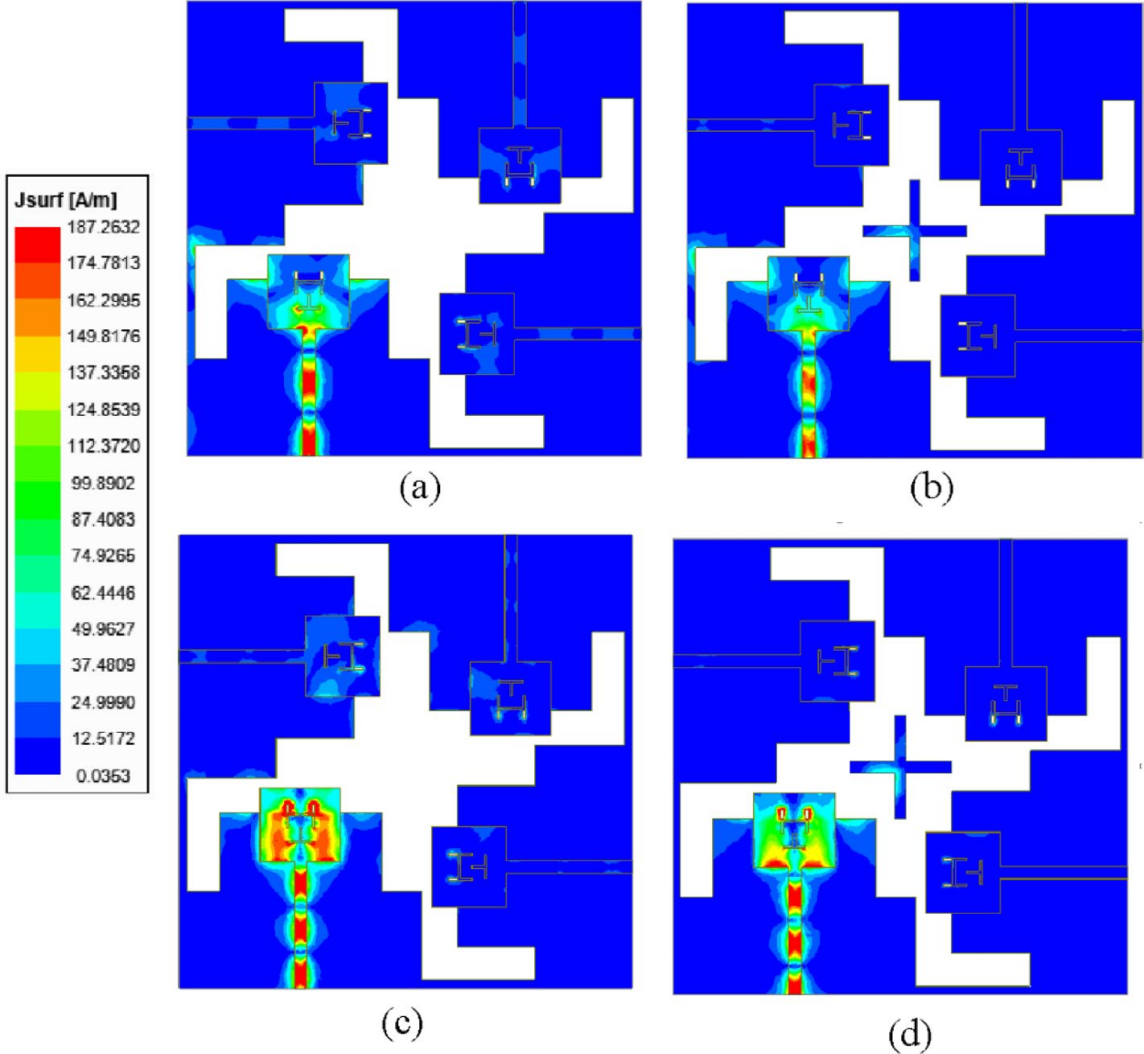
Surface current distributions of the proposed MIMO antenna at (**a**) 28 GHz without PSS, (**b**) 28 GHz with PSS, (**c**) 38 GHz without PSS, (**d**) 28 GHz with PSS.

Further, the surface current distributions at its two resonant frequencies (28 GHz and 38 GHz) are plotted under port P_1_ excitations without and with PSS in Fig. 9. It can be visualized form the figures that when the port P_1_ is excited without PSS structure, there are some surface currents present at the remaining ports, whereas when the PSS structure is introduced, negligible surface currents are induced at the other ports.

**Fig. 10:**
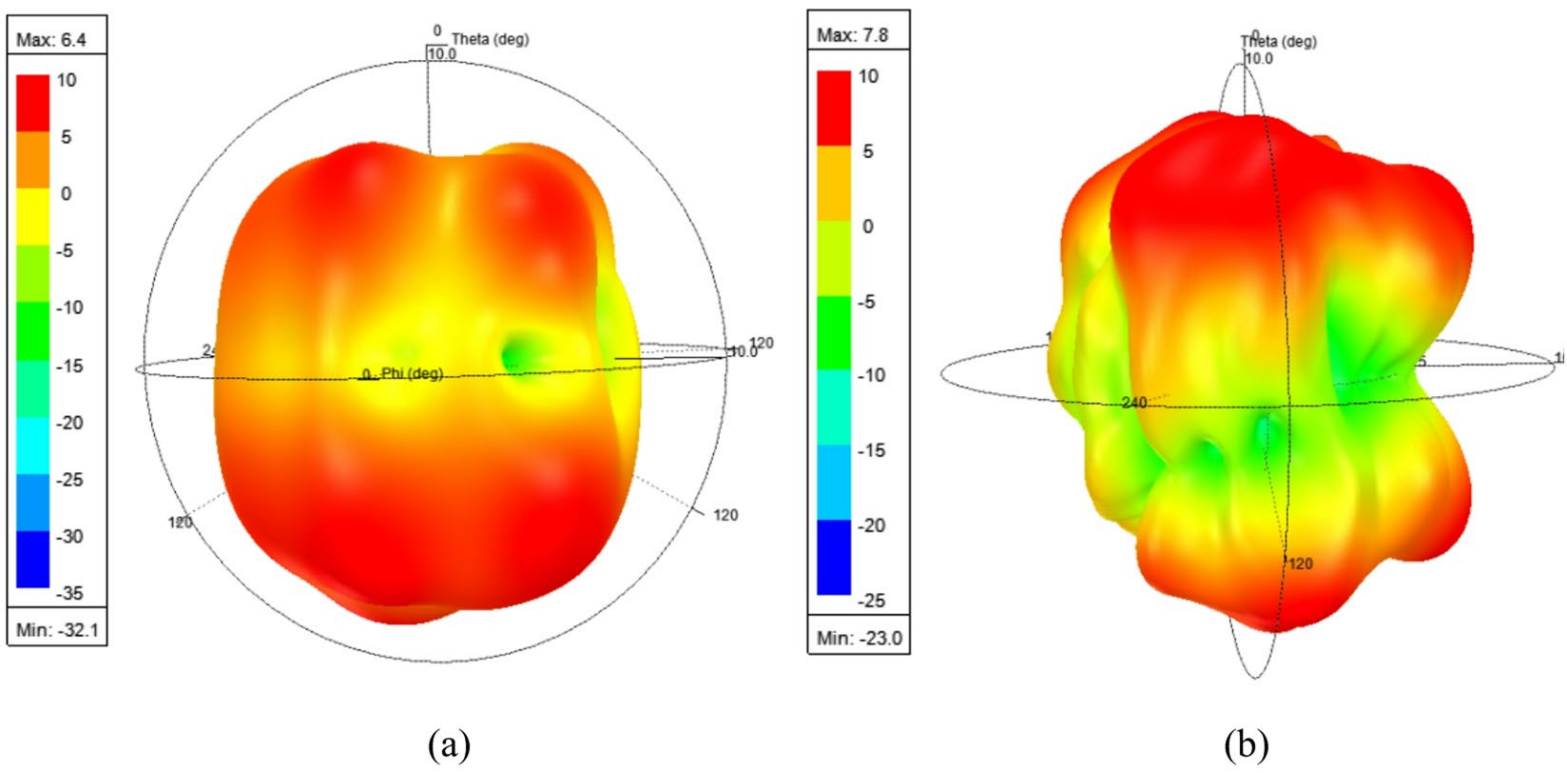
Simulated 3-D radiation patterns of the proposed MIMO antenna under port P_1_ excitation at (**a**) 28 GHz, (**b**) 38 GHz.

The 3-D radiation patterns of the designed MIMO antenna for both the operating frequencies of 28 GHz and 38 GHz, under port P_1_ excitation is shown in Fig. 10. The observed non-uniformity and the presence of nulls in the far-field patterns are mainly due to non-uniform surface current distribution and the increased electrical size of the antenna at millimeter-wave frequencies, which significantly influences the radiation behavior.

### Parametric analysis

**Fig. 11:**
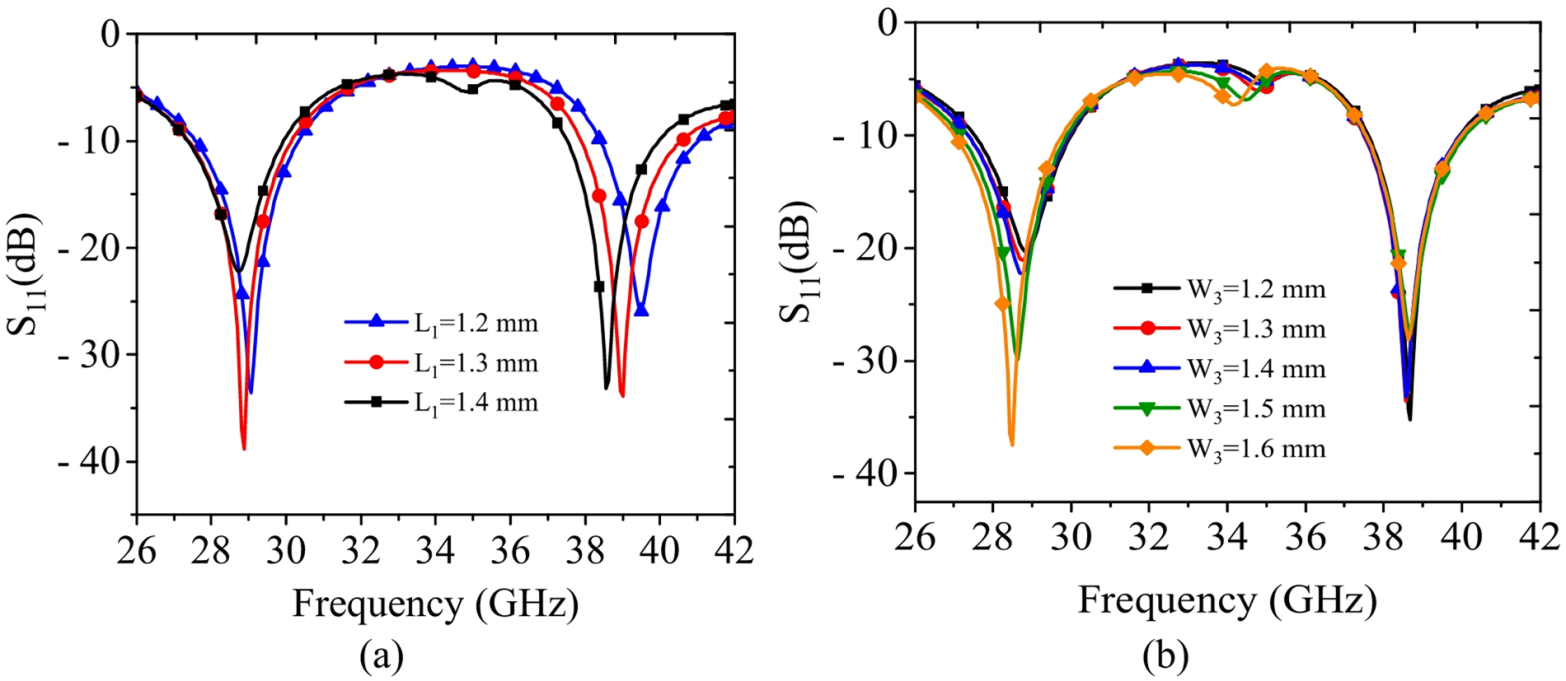
Simulated S_11_-parameters of different design parameters: (**a**) *L*_1_, (**b**) *W*_3_.

To analyze the influence of key design parameters on the S-parameters of the proposed antenna, a detailed parametric study of the critical geometrical parameters is carried out. During the parametric analysis, one parameter is varied at a time while all other parameters are kept constant. Figure 11(a) illustrates the S_11_-parameters for different values of length *L*_1_​. It can be observed that as *L*_1_​ increases, the higher operating frequency shifts toward lower frequencies, while the lower operating frequency is only slightly affected. Figure 11(b) shows the S-parameters for different values of *W*_3_​. As *W*_3_​ increases, the lower operating frequency shifts toward lower values, along with a noticeable widening of the lower band, while the higher operating frequency remains nearly unchanged. In a nutshell, the parametric study indicates that the lower operating frequency is primarily controlled by *W*_3_​, whereas the higher operating frequency is governed by *L*_1_​.

## Results and discussion

**Fig. 12:**
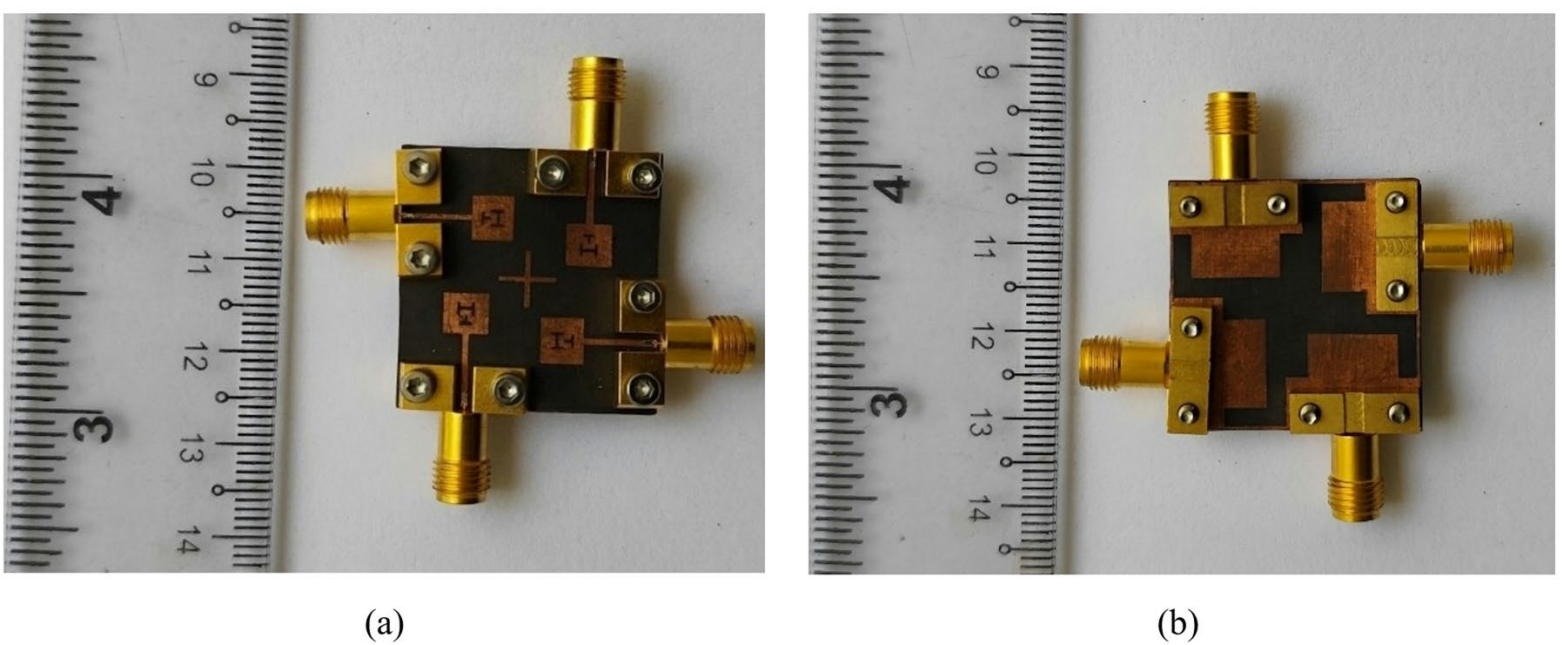
Fabricated prototype of the proposed dual-band MIMO antenna: (**a**) Top view, (**b**) Bottom view.

To validate the effectiveness of the proposed design methodology, the dual-band MIMO antenna is fabricated on the RT/Duroid 5880 substrate, as shown in Fig. 12, and its key performance parameters are experimentally measured and analyzed. For measurement purposes, four Rosenberger 02K243-40ME3 end-launch connectors are carefully mounted at the input ports of the antenna to facilitate S-parameter measurements and radiation pattern measurements. Mechanical screws are employed to assemble the structure to ensure uniform mechanical pressure, stable electrical grounding, and precise layer alignment, which are particularly critical at millimeter-wave frequencies due to the small wavelength.

**Fig. 13:**
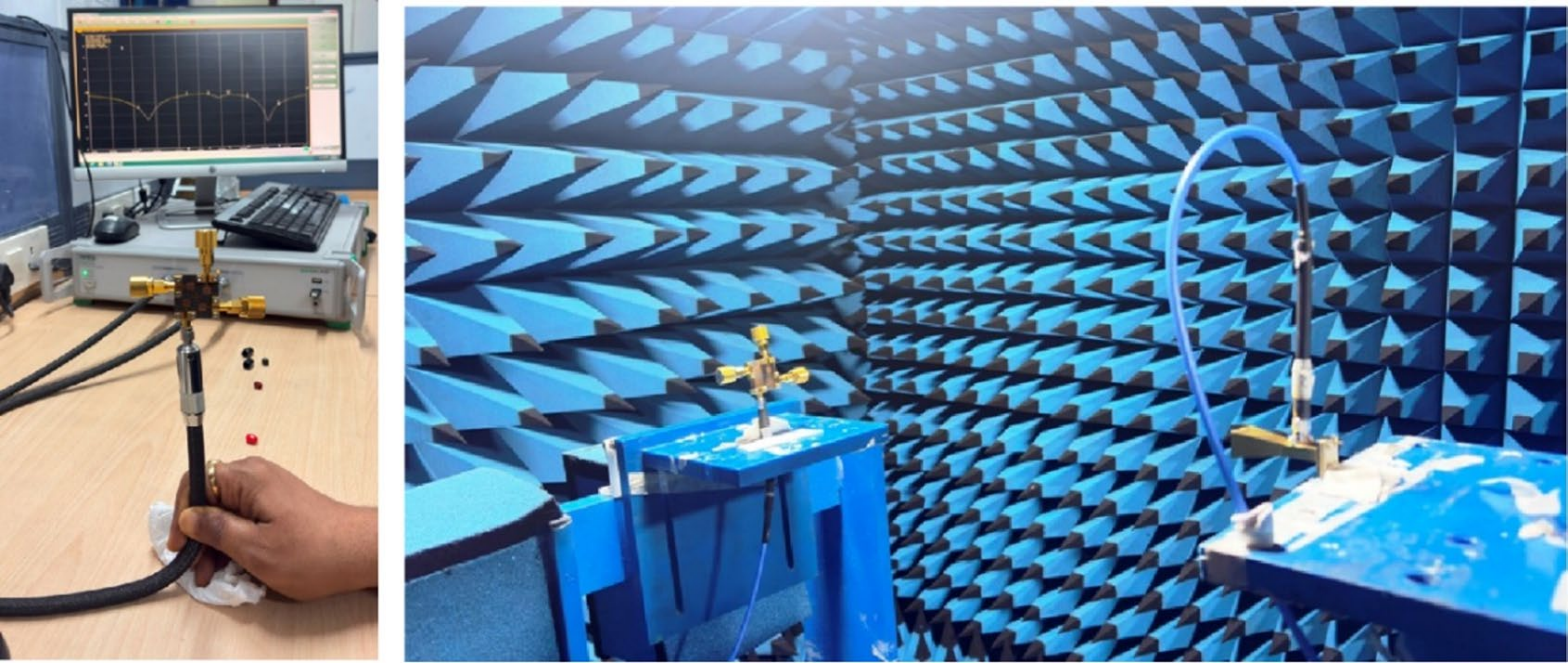
VNA setup for S-parameters measurements with anechoic chamber for radiation pattern measurements.

**Fig. 14:**
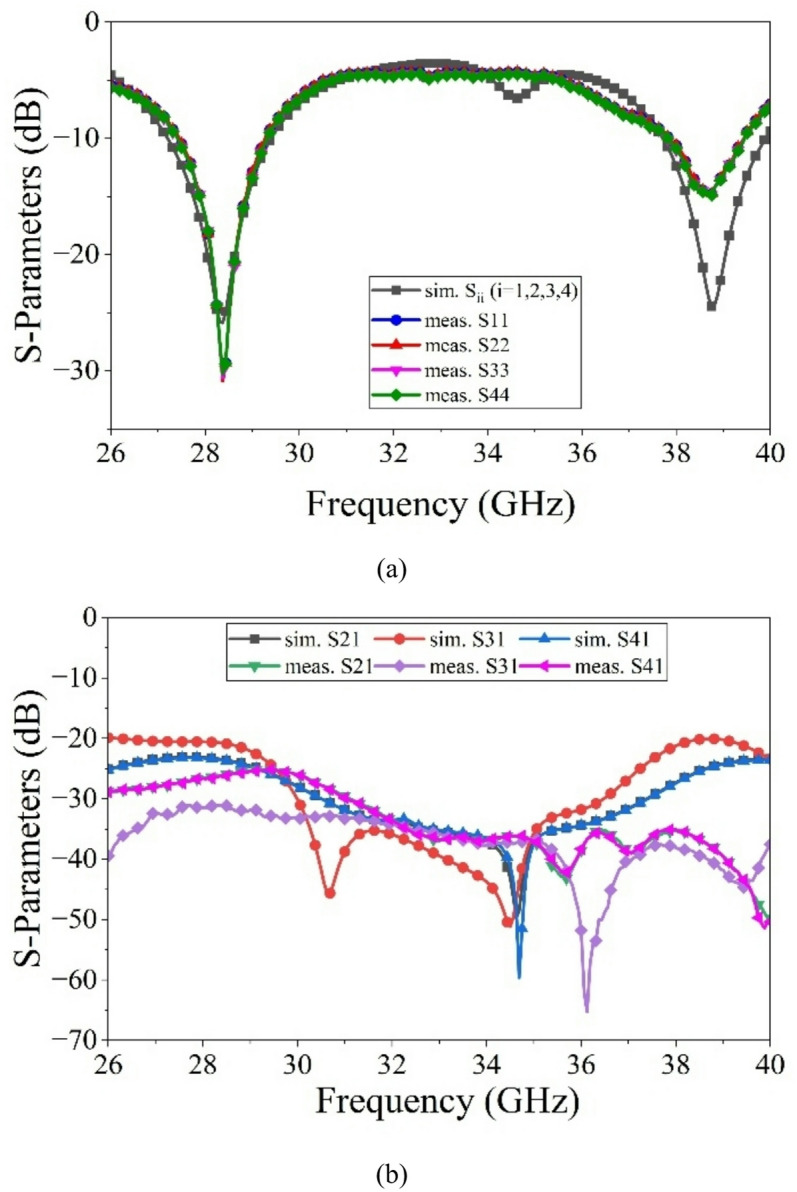
Simulated and measured S-parameters of the proposed dual-band MIMO antenna: (**a**) S_ii_, (**b**) S_ij_.

The VNA measurement set-up for S-parameters and anechoic chamber for radiation pattern measurements as shown in Fig. 13. A 43.5 GHz Anritsu MS46322B vector network analyzer (VNA) is used for S-parameter measurements. Whereas, the far-field radiation patterns are measured in an anechoic chamber of dimensions 6 m × 4 m × 4 m. The anechoic chamber is equipped with pyramidal RF absorbers providing reliable operation above 800 MHz. Figure 14(a) shows the simulated and measured S_ii_-parameters of the proposed MIMO antenna.

The designed antenna operates in the frequency bands 27.26–29.90 GHz and 37.57–40.29 GHz, with fractional bandwidths of 9.4% and 6.98%, respectively. The measured impedance bandwidths are approximately 26.75–29.37 GHz (FBW = 9.33%) and 37.56–39.87 GHz (FBW = 5.96%) around the center frequencies of 28 GHz and 38 GHz, respectively. Figure 14b presents the measured port-to-port isolations of the proposed dual-band MIMO antenna. The isolation levels exceed 19 dB and 22 dB for the lower and upper operating frequency bands, respectively, demonstrating effective mutual coupling reduction between antenna elements.

**Fig. 15:**
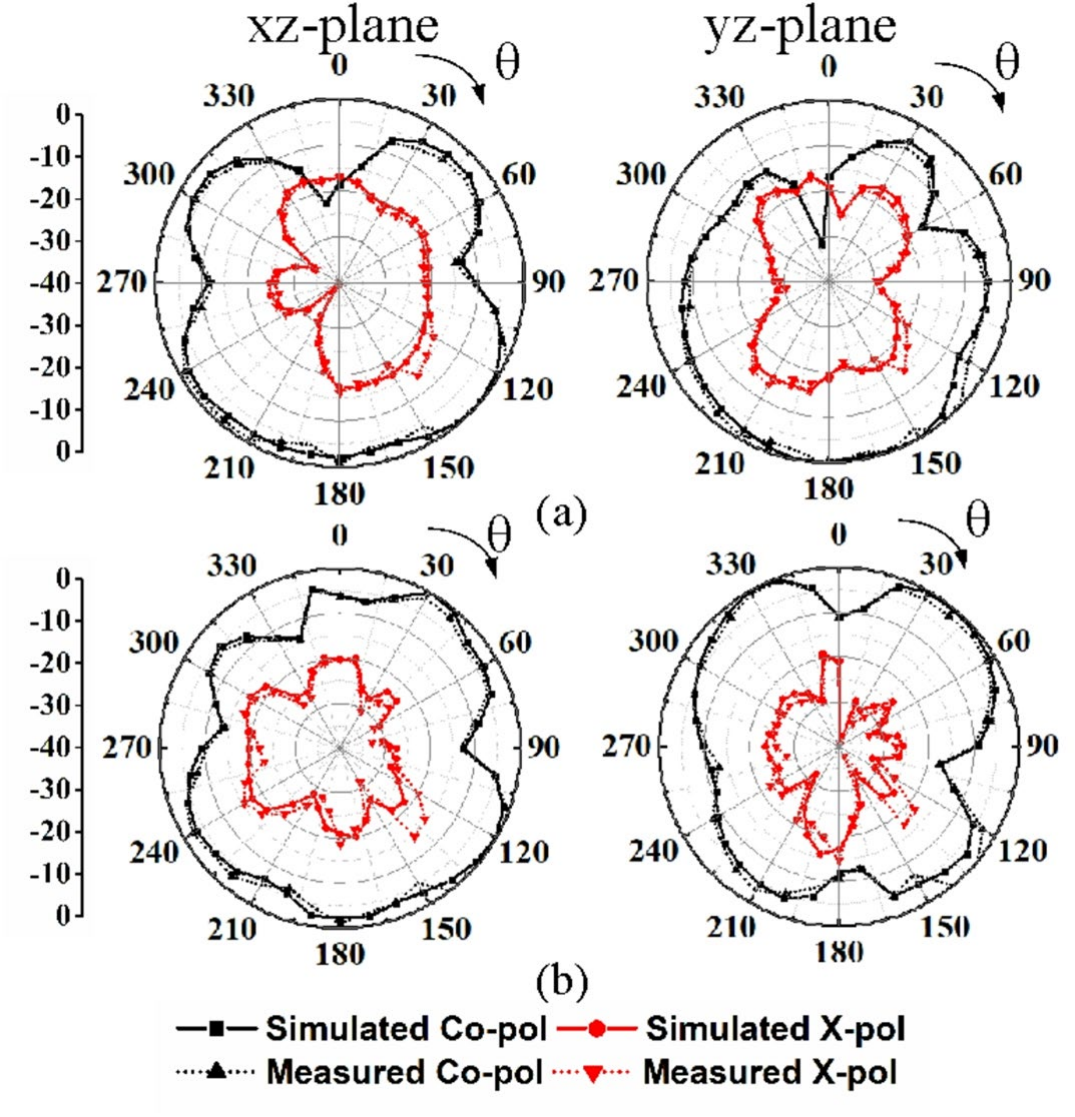
Simulated and measured far-field radiation patterns of the designed MIMO antenna under port P1 excitation: (**a**) 28 GHz, (**b**) 38 GHz.

**Fig. 16:**
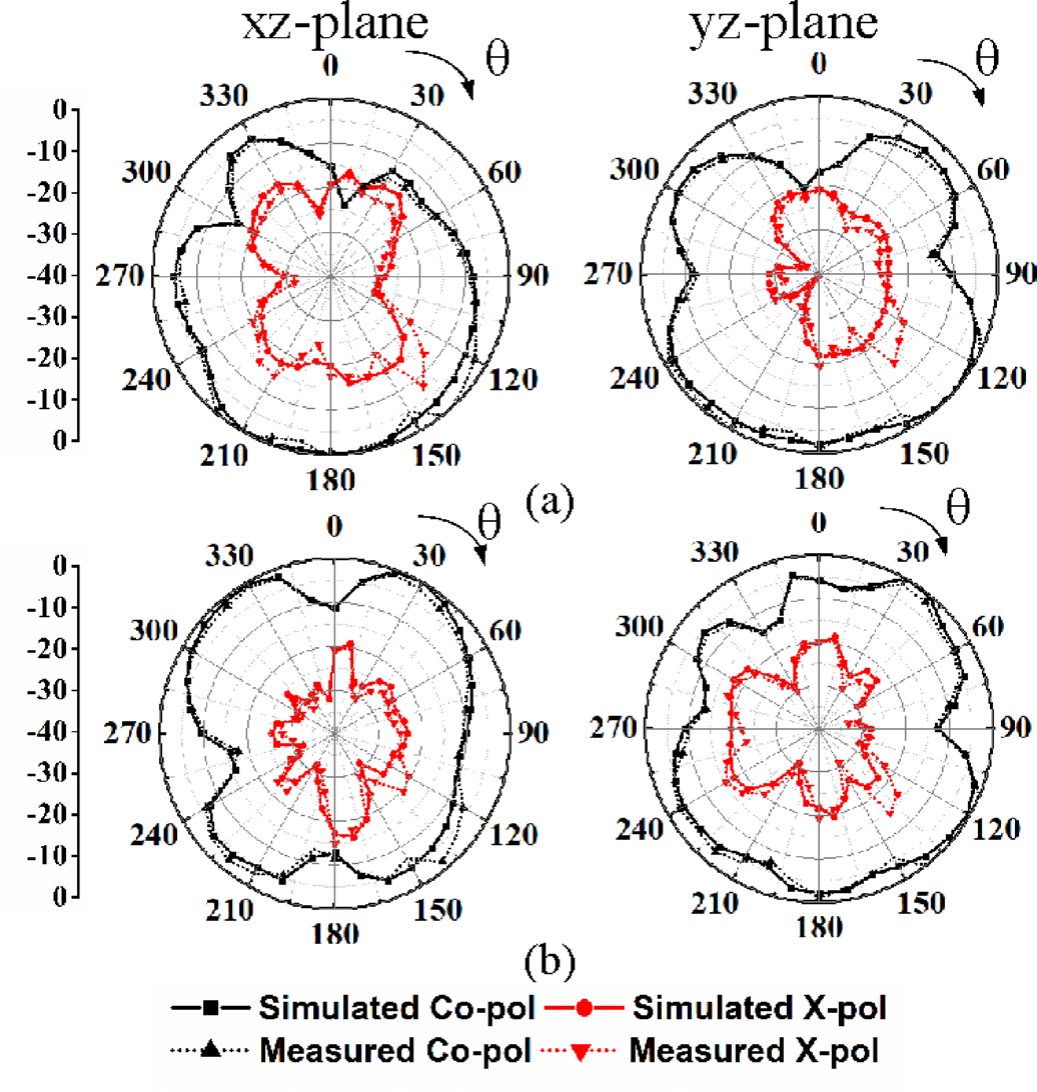
Simulated and measured far-field radiation patterns of the designed MIMO antenna under port P2 excitation: (**a**) 28 GHz, (**b**) 38 GHz.

Figures 15 and 16 illustrate the two-dimensional far-field radiation patterns of the proposed dual-band MIMO antenna at its two operating frequencies, 28 GHz and 38 GHz, in the *xz*-plane (E-plane) and *yz*-plane (H-plane), respectively. For brevity, the radiation patterns for only the first two ports excitations are presented. During the measurements, the unused ports are terminated with 50-Ω matched loads. The radiation patterns exhibit non-uniform omnidirectional characteristics at both operating frequencies, 28 GHz and 38 GHz. The non-uniform omnidirectional characteristics and the presence of nulls in the far-field radiation pattern can be attributed to the non-uniform surface current distribution, where the electrical dimensions of the antenna become significant. Phase variations across the radiating structure lead to destructive interference in certain angular directions, resulting in radiation nulls. Consequently, these combined electromagnetic effects lead to the observed deviations from ideal omnidirectional behavior.

**Fig. 17:**
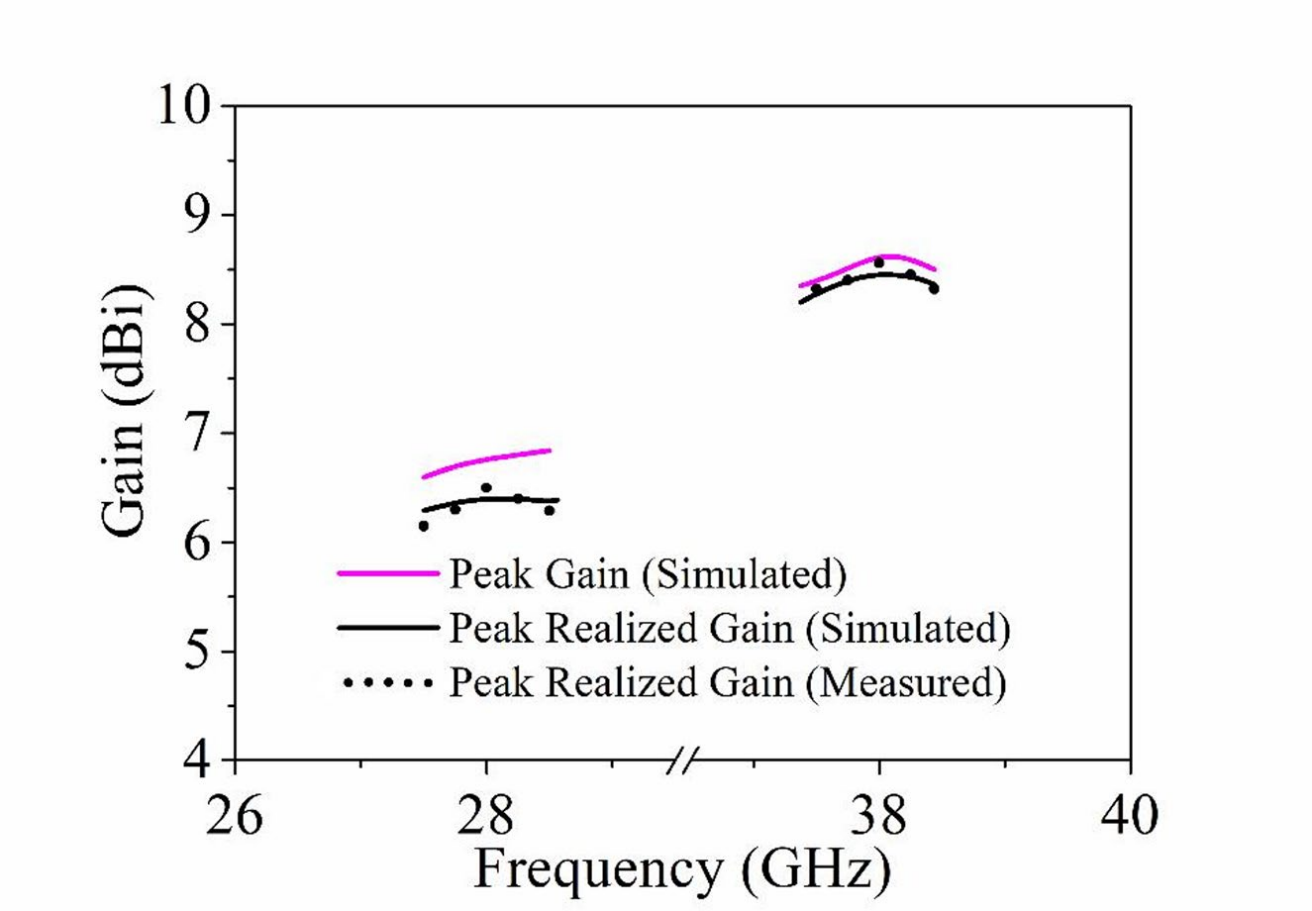
Simulated and measured gains of the proposed MIMO antenna.

**Fig. 18:**
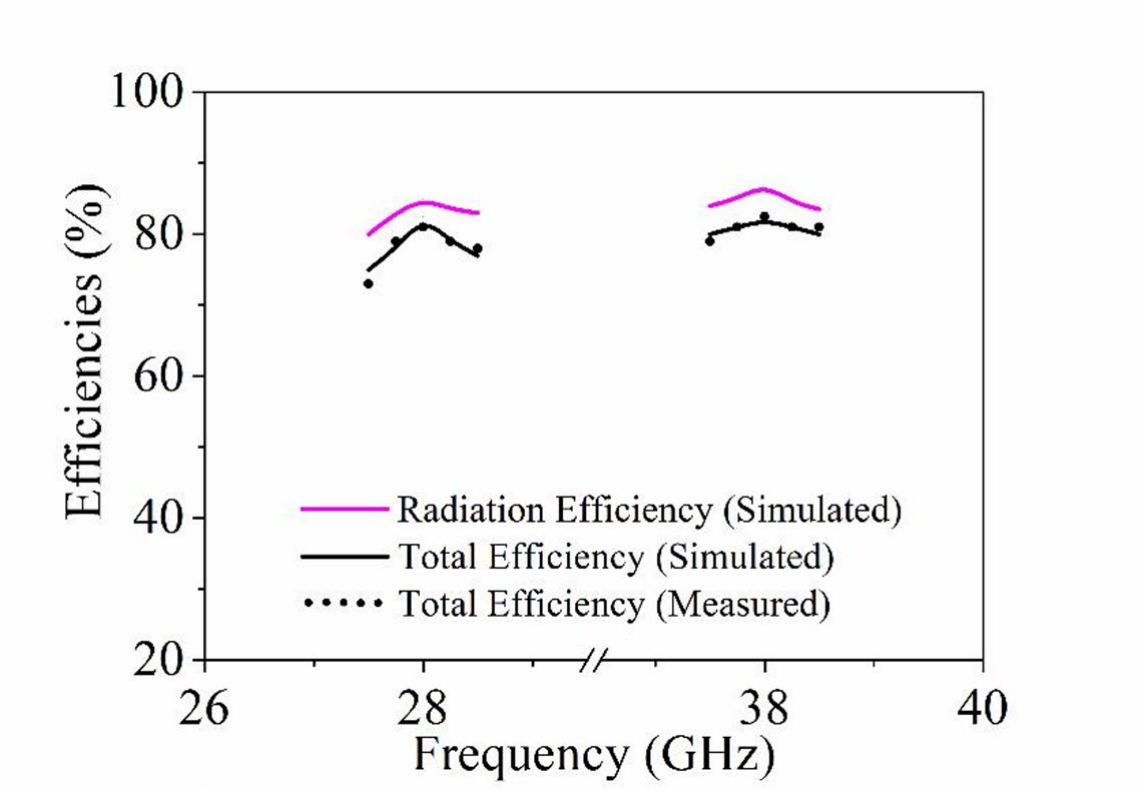
Simulated and measured efficiencies of the proposed MIMO antenna.

The peak realized gain of the proposed MIMO antenna is shown in Fig. 17, with values of 6.41 dBi and 8.50 dBi at the operating frequencies of 28 and 38 GHz, respectively. The total and radiation efficiencies of the designed four-port dual-band MIMO antenna over both operating frequencies (28 GHz and 38 GHz) are plotted in Fig. 18. It can be observed from the figure that the average radiation efficiencies are better than 80% over both the operating bands, while the total efficiencies are better than 75%.

## MIMO performance

To validate the effectiveness of the proposed antenna system in practical wireless communication scenarios, MIMO performance parameters are evaluated for ECC, diversity gain (DG), mean effective gain (MEG), channel capacity loss (CCL), and total active reflection coefficient (TARC).

### Envelope correlation coefficient (ECC)

In a MIMO system, ECC quantifies the correlation between the signals received by multiple antennas and serves as a critical parameter for evaluating diversity performance. ECCs $\left({\rho}_{ij}{\prime}s\right)$ are evaluated using the far-field radiation patterns of the antenna elements^23^, as described in Eq. (1).

1 $${\rho}_{ij}=\frac{{\left|{\iint{\stackrel{-}{E}}_{i}.\stackrel{-}{E}}_{j}^{*}d{\Omega}\right|}^{2}}{\iint{\left|{\stackrel{-}{E}}_{i}\right|}^{2}d{\Omega}.\iint{\left|{\stackrel{-}{E}}_{j}\right|}^{2}d{\Omega}}$$

**Fig. 19:**
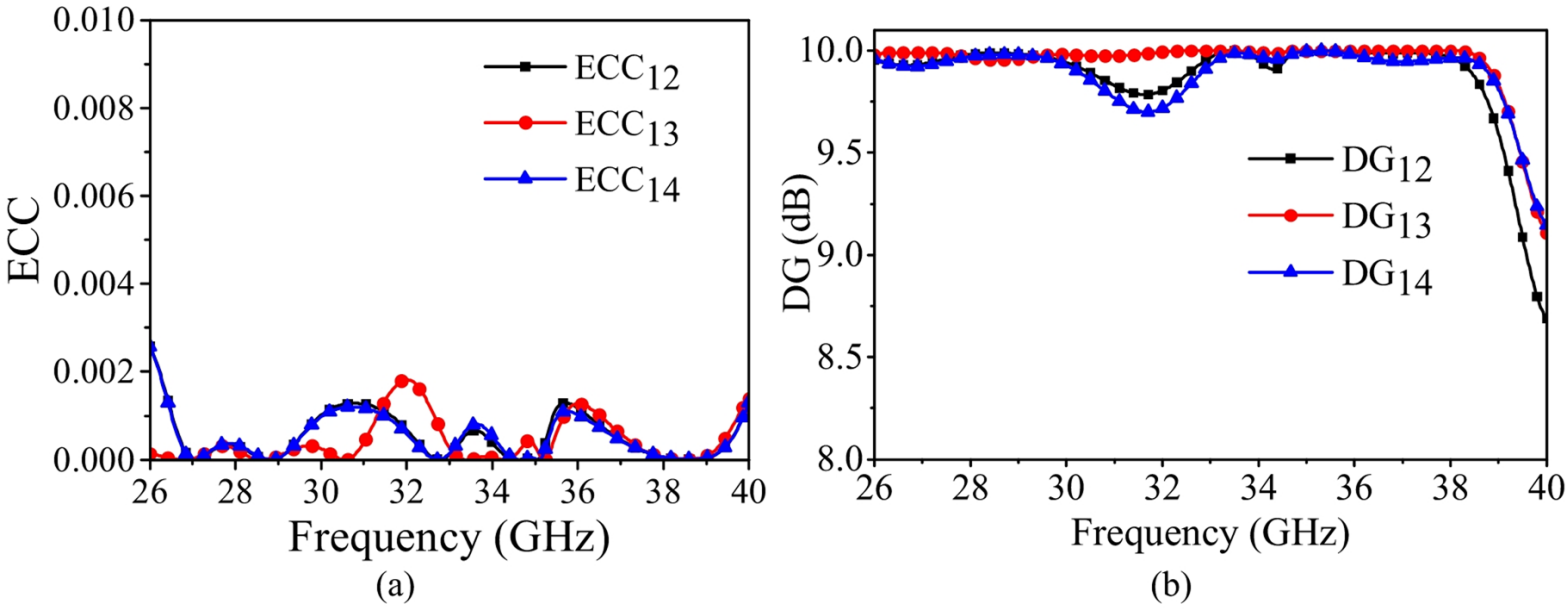
Performance of the proposed MIMO antenna: (**a**) ECC, (**b**) DG.

Figure 19a illustrates the ECC values of the proposed dual-band MIMO antenna, which remain within the acceptable limits (ECC < 0.0005) across both operating frequency bands. Such low ECC values indicate excellent isolation and minimal mutual coupling between the ports. This superior performance is primarily attributed to the orthogonal surface current distribution, the incorporation of the PSS, and the optimized ground plane configuration, which collectively suppress coupling fields and ensure pattern diversity.

### Diversity gain (DG)

DG quantifies the improvement in system reliability and robustness achieved through spatial diversity provided by multiple antennas and evaluates it using the ECCs Eq. (2)^24^.

2 $${DG}_{ij}=10\sqrt{1-{\left|{\rho}_{ij}\right|}^{2}}$$

Figure 19b illustrates the DG values of the proposed dual-band MIMO antenna. The DG values are found to be ~9.98-10 dB across the both the operating bands, which is very close to the ideal value of 10 dB. This confirms that the antenna system effectively exploits multipath propagation and provides strong diversity performance. The nearly ideal DG, combined with very low ECC, demonstrates that the proposed four-port MIMO antenna is well suited for high data rate millimeter-wave applications, ensuring reliable signal reception with reduced fading effects.

### Mean effective gain (MEG)

The diversity parameter MEG is also one of the important performance parameters in MIMO antenna systems because it directly reflects how effectively each antenna element receives (or contributes) power in a realistic multipath environment. The MEGs are evaluated using the following equation^25^.

3 $${MEG}_{i}=0.5[1-\sum_{j=1}^{4}{\left|{S}_{ij}\right|}^{2}]$$

**Fig. 20:**
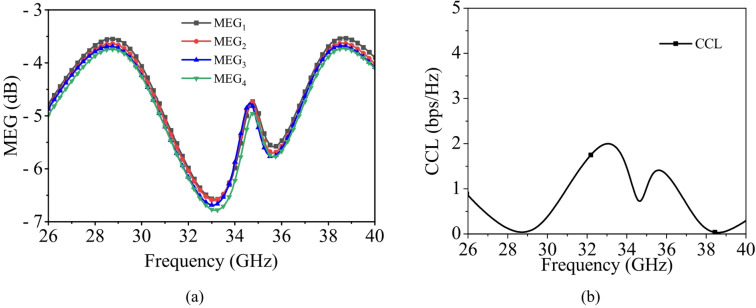
Performance of the proposed MIMO antenna: (**a**) MEG, (**b**) CCL.

Figure 20a presents the MEG values for the proposed MIMO antenna system, which remain within acceptable limits (MEG < -3 dB) across both the operating frequency bands.

### Channel capacity loss (CCL)

CCL measures the degree of signal degradation caused by the correlation between signals received by different antennas in a MIMO system. CCL is evaluated with the help of Eq. (3)^26^.

4 $$CCL=-{log}_{2}\left|{\mathrm{d}\mathrm{e}\mathrm{t}(\psi}^{R})\right|$$

where ${\psi}^{R}$ is the receive correlation matrix and is given as

5 $$\psi ^{R} = \left[ {\begin{array}{*{20}c} {\psi _{{11}} } & {\psi _{{12}} } & {\psi _{{13}} } & {\psi _{{14}} } \\ {\psi _{{21}} } & {\psi _{{22}} } & {\psi _{{22}} } & {\psi _{{24}} } \\ {\psi _{{31}} } & {\psi _{{32}} } & {\psi _{{33}} ~} & {\psi _{{34}} } \\ {\psi _{{41}} } & {\psi _{{42}} } & {\psi _{{43}} } & {\psi _{{44}} } \\ \end{array} } \right]$$

and $${\psi}_{ii}=1-\sum_{k=1}^{4}{\left|{S}_{ik}\right|}^{2}$$, $${\psi}_{ij}=-\sum_{k=1}^{4}{S}_{ik}{{S}_{jk}}^{*}$$ for $$i \ne j$$.

Figure 20(b) represents the CCL values for the proposed MIMO antenna system, which remain within acceptable limits (< 0.4 bps/Hz) across both the operating frequency bands.

### Total active reflection coefficient (TARC)

TARC measures the overall impedance matching of a MIMO antenna when all ports are excited simultaneously, accounting for mutual coupling and phase variations between signals. The TARC of the designed four port MIMO antenna is calculated as follows^27^:

6 $${\mathrm{T}}_{{\upchi}}=\sqrt{\frac{\sum_{m=1}^{4}{\left|{S}_{m1}+\sum_{n=2}^{4}{S}_{mn}{e}^{j{\theta}_{n-1}}\right|}^{2}}{4}}$$

7 $${\mathrm{T}}_{{\upchi}}=\sqrt{\frac{A+B+C+D}{4}}$$

where *A*,* B*,* C*, and *D* are the squared magnitudes of the signals with respective phase weights given as:

$$A = ~\left| {S_{{11}} ~ + ~S_{{12}} e^{{j\theta _{1} }} ~ + ~S_{{13}} e^{{j\theta _{2} }} ~~ + ~S_{{14}} e^{{j\theta _{3} }} } \right|^{2}$$

8 $$B = ~\left| {S_{{21}} ~ + ~S_{{22}} e^{{j\theta _{1} }} ~ + ~S_{{23}} e^{{j\theta _{2} }} ~~ + ~S_{{24}} e^{{j\theta _{3} }} } \right|^{2}$$

$$C = ~\left| {S_{{31}} ~ + ~S_{{32}} e^{{j\theta _{1} }} ~ + ~S_{{33}} e^{{j\theta _{2} }} ~~ + ~S_{{34}} e^{{j\theta _{3} }} } \right|^{2}$$

$$D = ~\left| {S_{{41}} ~ + ~S_{{42}} e^{{j\theta _{1} }} ~ + ~S_{{43}} e^{{j\theta _{2} }} ~~ + ~S_{{44}} e^{{j\theta _{3} }} } \right|^{2}$$.

**Fig. 21:**
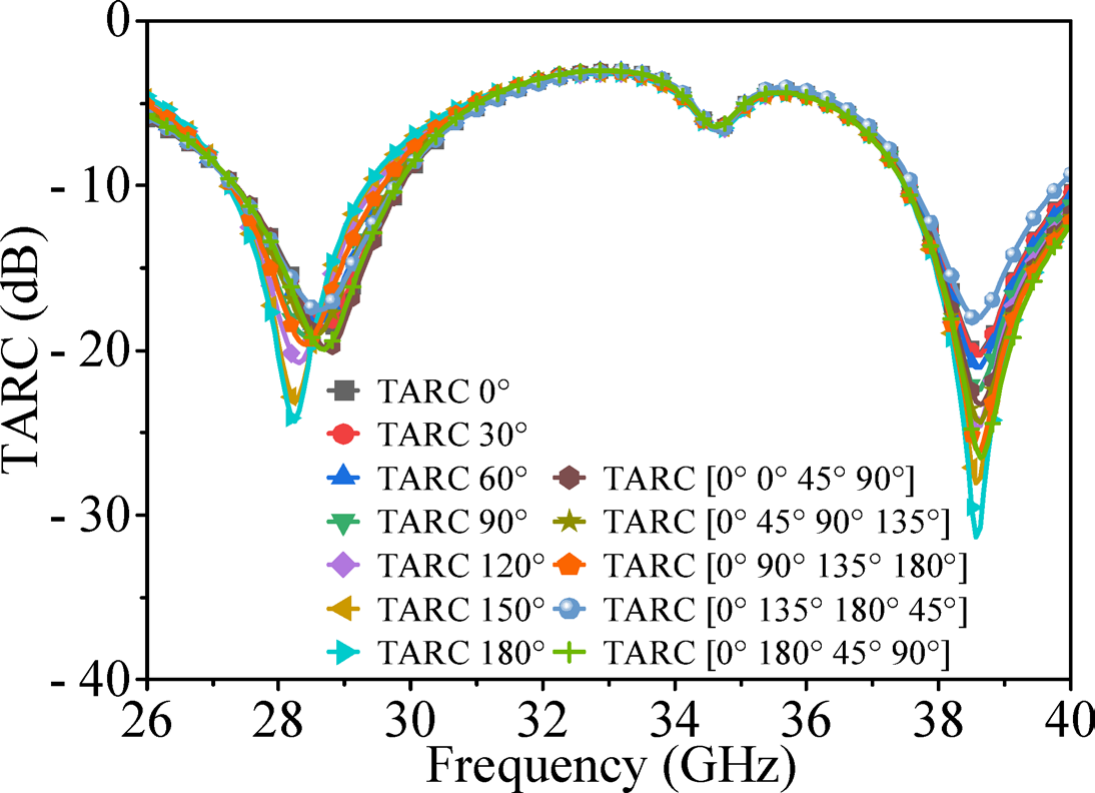
TARC of the proposed MIMO antenna.

Two excitation approaches are used to calculate TARC of the proposed MIMO antenna. In the first (uniform phase sweep), Port 1 was fixed at 0°, while Ports 2, 3, and 4 were excited simultaneously with identical phase values varied from 0° to 180° in 30° steps, representing balanced multiport excitation. In the second (non-uniform case), Port 1 remained at 0°, while the other ports were excited with different phase combinations: [0°, 45°, 90°], [45°, 90°, 135°], [90°, 135°, 180°], [135°, 180°, 45°], and [180°, 45°, 90°]. Figure 21 shows the TARC of the proposed MIMO antenna. The results show that TARC remains largely independent of excitation phase and stays below −10 dB across both the operating band of the designed MIMO antenna, indicating good impedance matching, low active reflections, and effective decoupling among antenna elements for reliable MIMO performance.

**Fig. 22:**
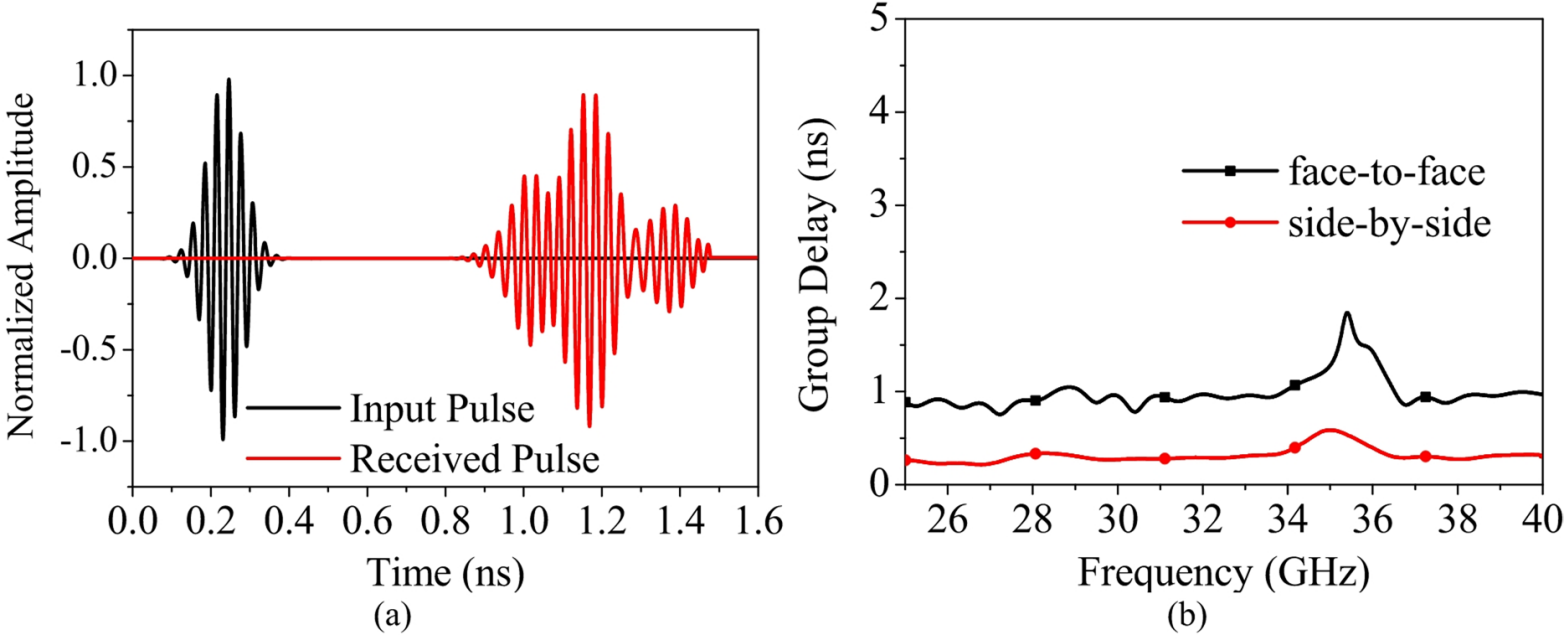
(**a**) Input and received signals, (**b**) Group delay response of the proposed identical antennas placed 30 cm apart in face-to-face and side-by-side configurations.

### Time domain analysis

A fidelity factor close to 1 and a group delay response with minimal variation are required to ensure consistent signal characteristics and distortion free performance from the designed antenna. The fidelity factor can be defined as^28^:

9 $$F={max}_{\tau}\left[\frac{\underset{-\infty}{\overset{+\infty}{\int}}{s}_{t}\left(t\right){s}_{r}\left(t+\tau\right)dt}{\underset{-\infty}{\overset{+\infty}{\int}}{s}_{t}^{2}\left(t\right)dt\underset{-\infty}{\overset{+\infty}{\int}}{s}_{r}^{2}\left(t\right)dt}\right]$$

where ${s}_{t}\left(t\right)$ and ${s}_{r}\left(t\right)$ are the input and output signals. Figure 22(a) shows the input and received signals, achieving a face-to-face fidelity factor of 0.92, which indicates high waveform similarity and low signal distortion. The group delay response shown in Fig. 22(b) remains stable, exhibiting an average variation less than 2 ns across both the operating bands. These group delay characteristics confirm that the proposed antenna maintains good phase linearity across its operating resonant frequency range.

The proposed four-port dual-band MIMO antenna demonstrates excellent performance, validating its ability to support reliable, high-capacity, and low-latency communication, making it a strong candidate for next-generation 5G millimeter-wave applications.

## Performance comparison

**Table 1:** Comparison with other reported millimeter wave dual-band MIMO antennas

Ref	No. of ports	Overall Size (mm^3^)	Operating Bandwidth (GHz)	IBW (GHz)	Isolation (dB)	ECC	Peak Gain (dBi)
^16^	2	7.5 × 8.8 × 0.25	28, 38	1.23, 1.06	> 20	< 0.01	6.6, 5.86
^17^	2	19 × 29 × 13	26, 38	1.7, 1.9	> 30, > 22	< 0.001	9.5 11.4
^18^	4	18 × 8.5 × 0.25	27.76–28.48, 37.69–38.19	0.72, 0.5	> 20	< 0.03	4.15, 7.73
^19^	4	26 × 26 × 0.25	27.7–28.3, 37.7–38.3	0.6, 0.6	> 33	< 0.0005	7.4, 8.1
^20^	4	28 × 28 × 0.508	26.6–29.3, 37.6–38.35	2.7, 0.75	> 32	< 0.0005	7, 7.9
^21^	4	17.76 × 17.76 × 1.52	27.66–28.378, 37.7–38.26	0.718, 0.56	> 68, > 90	< 3 × 10^−5^	7.37, 8.13
^22^	4	24.2 × 29 × 0.203	27.5–29.3, 37.5–38.2	1.8, 0.7	≥ 20	≤ 0.001	8.1, 7.22
**Prop**	**4**	**28** × **28** × **0.254**	**27.26–29.90,** **37.57–40.29**	**2.64,** **2.72**	** > 23,** ** > 27**	** < 1 × 10** ^**−3**^	**6.41,** **8.50**

## Conclusion

In this work, a compact four-port dual-band MIMO antenna has been designed and developed for 5G NR FR-2 spectrum. The proposed antenna operates over 26.9–28.83 GHz and 37.04–39.48 GHz, effectively covering the n257/n261 and n260 bands of the 5G NR FR2 spectrum. The designed antenna achieves inter-port isolation better than 20 dB across both bands, with an overall compact volume of 28 mm × 28 mm × 0.254 mm (≈$0.161{\lambda}_{0}^{3}$ at 28 GHz). The MIMO system’s performance, evaluated using parameters such as the ECC, DG, MEG, CCL, and TARC, remains within acceptable thresholds. These results confirm that the designed antenna provides reliable, high-capacity, and low-latency communication, making it a strong candidate for integration into next-generation 5G millimeter-wave communication systems.

## Data Availability

The datasets used and/or analyzed during the current study are available from the corresponding author on reasonable request.
